# An Assessment of the Genotoxicity and Subchronic Toxicity of a Supercritical Fluid Extract of the Aerial Parts of Hemp

**DOI:** 10.1155/2018/8143582

**Published:** 2018-06-07

**Authors:** Tennille K. Marx, Robin Reddeman, Amy E. Clewell, John R. Endres, Erzsébet Béres, Adél Vértesi, Róbert Glávits, Gábor Hirka, Ilona Pasics Szakonyiné

**Affiliations:** ^1^AIBMR Life Sciences, Inc., 2800 E Madison St., Suite 202, Seattle, WA 98112, USA; ^2^Toxi-Coop Zrt., Magyar Jakobinusok Tere 4/B, Budapest 1122, Hungary

## Abstract

A battery of toxicological studies was conducted on a supercritical CO_2_ extract of the aerial parts of the* Cannabis sativa *plant, containing approximately 25% cannabinoids. No evidence of genotoxicity was found in a bacterial reverse mutation test (Ames), in an in vitro mammalian chromosomal aberration test, or in an in vivo mouse micronucleus study. A 14-day repeated oral dose-range finding study conducted in Wistar rats at 1000, 2000, and 4000 mg/kg bw/day resulted in effects where a NOAEL could not be concluded. Based on those results, a 90-day repeated dose oral toxicity study was performed in rats using doses of 100, 360, and 720 mg/kg bw/day, followed by a 28-day recovery period for two satellite groups. Significant decreases in body weight, body weight gain, and differences in various organ weights compared to controls were observed. At the end of the recovery period, many of the findings were trending toward normal; thus, the changes appeared to be reversible. The NOAEL for the hemp extract in Hsd.Han Wistar rats was considered to be 100 mg/kg bw/day for males and 360 mg/kg bw/day for females.

## 1. Introduction

*Cannabis sativa *L. is a unique and complex plant with respect to its constituents and physiological properties, some of which have opposing effects [1]. Despite the fact that humans have utilized the* C. sativa *plant medicinally for millennia, its chemical profile and complex pharmacology have yet to be fully elucidated [1–3]. One group of constituents that has been researched is the cannabinoids—oxygen-containing aromatic hydrocarbon compounds that constitute at least 70 of the estimated 400+ constituents in the plant (e.g., cannabichromene, cannabielsoin, cannabicyclol, and cannabidiol) [4–6]. Delta-9-tetrahydrocannabinol (THC) is the most recognized cannabinoid in certain strains of* C. sativa* due to its well-known psychotropic properties. However, the cannabinoid that is most concentrated in the test article utilized in the present set of studies is cannabidiol (CBD), which is nonintoxicating and nonsedating, and according to Russo (2017) there is no compelling evidence that CBD undergoes cyclization or bioconversion to THC in humans [7].

Cannabinoids are chiefly known to act on the cannabinoid receptors CB1 and CB2 (as well as transient potential vanilloid channel type 1 receptors) [8, 9]. CB1 receptors are primarily found in the central nervous system but are also found in peripheral tissues, including those of the pituitary gland, gastrointestinal system, reproductive system, and immune system [8, 10]. CB2 receptors are found in the central nervous system (e.g., neuronal microglia cells, brain stem cells, and cerebellum) as well as peripherally in tissues such as the spleen, thymus, tonsils, mast cells, and reproductive system [8, 11, 12].

Two recent reviews on the safety and side effects of CBD concluded that CBD appears to have a favorable safety profile in humans according to the scientific literature—for example, it does not seem to induce changes in food intake, affect physiological parameters such as heart rate, blood pressure, and body temperature and does not affect gastrointestinal transit or alter psychomotor or psychological functions, even with chronic use in humans at doses of 600–1,500 mg/day [13, 14]. However, the authors concluded that more chronic human studies are needed for evaluating the potential side effects of CBD, as the number of individuals in many clinical trials was small, and more aspects of toxicological evaluations (such as genotoxicity studies and further animal studies) are still needed.

Indeed, there is an overall lack of published oral toxicological studies meeting current international standards on CBD, hemp, or hemp extracts from the aerial parts of* C. sativa*. We are aware of only one published CBD oral toxicity study, a 90-day repeated-dose study conducted by Rosenkrantz et al. (1981) in Rhesus monkeys [15]. This study was conducted prior to the adoption of Organisation of Economic Cooperation and Development (OECD) guidelines for 90-day repeated dose oral toxicity studies (1981) and OECD Good Laboratory Practice (GLP) standards (1992). Four monkeys/sex/group received nearly pure CBD by gavage at doses of 30, 100, and 300 mg/kg bw/day for 90 days. The study results showed no clear dose-dependent toxicologically relevant changes, except for significantly lower relative testicular-to-brain weights in the high-dose group and inhibition of spermatogenesis in all treated male monkeys. Limitations of the study include the involvement of male monkeys at various stages of sexual maturity and unreported ages of the animals.

Herein we report on a battery of OECD-compliant toxicological studies—a bacterial reverse mutation test, in vitro mammalian chromosomal aberration test, in vivo mouse micronucleus test, and 14-day and 90-day repeated dose oral toxicity studies—conducted on a supercritical CO_2_ extract of the aerial parts of hemp in order to investigate its potential genotoxicity and subchronic toxicity in rats. To further investigate CBD's effects on the male genitourinary system (performed due to the results noted in Rosenkrantz et al.'s study described above), the 90-day repeated dose oral toxicity study included a quantitative and qualitative sperm analysis (spermatids, sperm motility, and morphology).

## 2. Materials and Methods

### 2.1. Test Article

CV Sciences, Inc. (San Diego, CA) supplied the test article, a proprietary supercritical CO_2_ extract of the aerial parts of hemp (*C. sativa*). Certified growers in Europe harvest the* C. sativa* and dry the raw materials (aerial parts) of the plant, which are then processed via a critical CO_2_ extraction to obtain the oil. Edible fatty acids comprise 61% of this concentrated extract, while phytocannabinoids are present at 26% (of this, approximately 96% is CBD and less than 1% is THC); the remaining 13% include fatty alkanes, plant sterols, triterpenes, and tocopherols and thus approximately 100% of the extract constituents are accounted for. Newly developed analytical methods and testing since the time these studies were performed have also consistently revealed low levels of other phytocannabinoids (e.g., cannabichromene, cannabigerol, cannabicyclol, and cannabinol) in subsequent batches of this natural extract of the aerial parts of hemp. The tests reported below were conducted according to GLP and OECD guidelines and as previously described by Clewell et al. [16].

### 2.2. In Vitro Studies

#### 2.2.1. Bacterial Reverse Mutation Test

The mutagenic potential of the test article was evaluated in a bacterial reverse mutation test using* Salmonella typhimurium *(TA98, TA100, TA1535, and TA1537) and* Escherichia coli *WP2*uvrA* (Moltox, Inc., Boone, NC) in the presence and absence of activated rat liver S9 (Moltox, Inc., Boone, NC). The study was performed following methods previously described by Ames et al. [17], Maron and Ames [18], Kier et al. [19], and Venitt and Parry [20] and according to OECD Guideline No. 471 (1997) [21], Environmental Protection Agency (EPA) Guideline Office of Prevention, Pesticides and Toxic Substances (OPPTS) 870.5100 (1998), European Commission (EC) No. 440/2008 [22], and International Conference on Harmonisation (ICH) Guidance S2(R1) (2012) [23].

Based on a preliminary solubility test and a preliminary range finding test, seven concentrations, 5, 16, 50, 160, 500, 1600, and 5000 *μ*g/plate, were selected for the initial and confirmatory tests. Formulations were prepared by dissolving the test article in dimethyl sulfoxide (DMSO). The following strain specific positive controls, for the experiments without metabolic activation, were used to demonstrate the effectiveness of the test: 4-Nitro-1,2-phenylenediamine (NPD) (4 *μ*g/plate) was used for TA98, sodium azide (SAZ) (2 *μ*g/plate) for TA100 and TA1535, 9-aminoacridine (9-AA) (50 *μ*g/plate) for TA1537, and methyl methanesulfonate (MMS) (2 *μ*g/plate) for WP2. The positive control for experiments with metabolic activation was 2-aminoanthracene (2-AA) (2 *μ*g/plate and 50 *μ*g/plate for all* S. typhimurium* strains and the* E. coli* WP2*uvrA *strain, resp.). Two negative (vehicle) control groups were utilized because of the different solubility of the test article and positive control items. DMSO served as the vehicle control for the test article, NPD, 9-AA, and 2-AA and ultrapure water (ASTM type 1, prepared by Direct-Q5 system, Millipore) for SAZ and MMS.

A standard plate incorporation procedure was used for the initial mutation test. Tester strains were exposed to the test article at each concentration and to positive and negative controls, both with and without S9 metabolic activation, and plates were incubated for 48 hours at 37°C. The confirmatory mutation test was conducted using a 20-minute preincubation procedure prior to plating and another 48-hour incubation period after plating at 37°C. All experiments were conducted in triplicate.

Colony numbers were determined by manual counting, from which mean values, standard deviations, and mutation rates were calculated. A result was considered positive if a dose related increase in revertant colonies occurred and/or a reproducible biologically relevant positive response for at least one dose group occurred in at least one strain with or without metabolic activation. A result was considered biologically relevant if the increase was twice that of negative controls for strain TA100 or if the increase was three times that of negative controls for all other strains.

#### 2.2.2. In Vitro Mammalian Chromosomal Aberration Test

An in vitro mammalian chromosomal aberration test was performed to determine whether the test article could induce structural chromosomal aberrations in cultured V79 Chinese hamster lung cells. It was performed in compliance with internationally accepted guidelines: OECD 473 (2014) [24], EC No. 440/2008 [22], and US EPA OPPTS 870.5375 (1998) [25].

Solubility and cytotoxicity of the test article were assessed for the purpose of selecting concentrations for the main test. Two independent experiments were conducted in the main test. In Experiment A, V79 cultures (5 × 10^5^ cells/group) were exposed to the negative control or each test article concentration for a three-hour period with (50, 70, and 90 *μ*g/mL) and without (10, 20, and 30 *μ*g/mL) metabolic activation. Groups of cells were also exposed to the respective positive controls (ethyl methanesulfonate and cyclophosphamide). Following the exposure period, the cells were washed with Dulbecco's Modified Eagle's Medium containing 5% fetal bovine serum, and growth medium was added. Sampling was made 20 hours following the start of treatment. All individual test article and negative and positive control experiments were carried out in duplicate, and the Relative Increase in Cell Counts was calculated.

Experiment B was conducted as described for Experiment A except that the exposure period without metabolic activation was 20 hours (while exposure with metabolic activation remained 3 hours), and sampling was made after 20 hours for groups treated without metabolic activation and after 28 hours (to cover the potential for mitotic delay) for groups treated both with and without metabolic activation. The test article concentrations were 50, 70, and 90 *μ*g/mL with S9 metabolic activation and 1.25, 2.5, and 5 *μ*g/mL without activation.

Chromosomes were treated with colchicine (Sigma-Aldrich Co.) (0.2 *μ*g/mL) for 2.5 hours followed by harvesting, swelling with 0.075M KCl, and washing in fixative for approximately 10 minutes before preparing slides, air-drying, and staining with 5% Giemsa (Merck & Co., Inc.). At least 400 metaphase cells from each experimental group, containing 22 ± 2 centromeres, were evaluated for structural aberrations (slides were coded and scored blind). Chromatid and chromosome type aberrations (gaps, deletions, breaks, and exchanges) were recorded separately. Polyploid and endoreduplicated cells were also scored. Nomenclature and classification of chromosomal aberrations were based on publications by International System for Human Cytogenetic Nomenclature [26] and Savage [27]. Fisher's exact test and *χ*^2^ test were utilized for statistical analysis. The test article was considered as nonclastogenic if there were no statistically significant increases in the number of metaphases with aberrations in dose groups compared to the negative control and/or if the number of metaphases with aberrations was within the range of the laboratory's historical control data.

### 2.3. Animal Studies

Care and use of study animals were in compliance with laboratory standard operating procedures under the permission of the Toxi-Coop Zrt. Institutional Animal Care and Use Committee. The 14-day and 90-day studies are also performed in accordance with the National Research Council Guide for Care and Use of Laboratory Animals [28] and in compliance with the principles of the Hungarian Act 2011 CLVIII (modification of Hungarian Act 1998 XXVIII) regulating animal protection. Animals received ssniff® SM R/M-Z+H complete diet (Experimental Animal Diets, Inc., Soest, Germany) and potable tap water* ad libitum*.

#### 2.3.1. In Vivo Mouse Micronucleus Test

The genotoxic potential of the test article was further assessed in an in vivo mouse micronucleus test. The study was conducted in compliance with OECD 474 (2014) [29], EC No. 440/2008 [22], and US EPA OPPTS 870.5395 (1998) [30].

Specific pathogen-free Crl:NMRI BR mice aged eight weeks and with body weights of 32.6–36.4 g were utilized for the study. They were acclimatized for eight days and housed two animals per cage in the pretest and 5–7 animals per cage in the main test. Housing conditions were 22 ± 3 °C, 30–70% relative humidity, and a 12-hour light-dark cycle.

Humaqua (sterile water, TEVA Pharmaceutical Works Private Ltd., Co.) was used as the negative control and as the vehicle for administration of the positive control (cyclophosphamide (Sigma-Aldrich, Germany)). Sunflower oil was also used as a negative control, as well as the solvent for the test article. Test article was prepared within two hours of administration at concentrations of 50, 100, and 200 mg/mL.

A preliminary toxicity test was conducted to determine the appropriate high dose for the main test and whether there were large differences in toxicity between sexes. A single dose of the test article was administered by gavage to two male and female mice at a concentration of 2000 mg/kg body weight (bw), and the animals were observed at regular intervals for signs of toxicity and mortality.

On the basis of the results of the preliminary toxicity test, single oral gavage doses of 500 (*n* = 5), 1000 (*n* = 5), and 2000 (*n* = 10) mg/kg bw were chosen for the main study. Male Crl:NMRI BR mice were randomly divided into five groups: a negative control (*n* = 10), positive control (*n* = 5), and the three test groups. The positive control, cyclophosphamide 60 mg/kg bw, was given intraperitoneal injection. Two extra animals were included in the high-dose group in order to maintain statistical power in case any animals died before the scheduled sacrifices. In the case of no premature deaths, bone marrow slides were not prepared from the extra animals.

All animals were observed immediately after dosing and at regular intervals until sacrifice (by cervical dislocation) for visible signs of reactions to treatment. In the positive control, low- and mid-dose groups, the sacrifices were made at 24 hours after treatment. In the high-dose and negative-control groups, sacrifices were made at 24 and 48 hours after treatment (five animals were used for sampling on each occasion). Bone marrow smears were prepared on standard microscope slides from two exposed femurs of the mice from every time point immediately after sacrificing. Four thousand polychromatic erythrocytes (PCEs) per animal were scored for the incidence of micronucleated PCEs (MPCEs). The proportion of immature among total erythrocytes was determined per animal by counting a total of at least 500 immature erythrocytes.

Statistical analysis was performed using Kruskal-Wallis nonparametric Analysis of Variance (ANOVA) test. A positive response was defined as a statistically significant increase in the frequency of MPCEs (compared to negative controls) in at least one sampling time that was dose-related and outside laboratory historical control ranges.

#### 2.3.2. 14-Day Repeated Dose Oral Toxicity Study

A 14-day repeated dose oral toxicity study in healthy 49–52-day-old Hsd.Han Wistar rats was conducted in order to obtain information on the toxic potential of the test article in male and female rats over a 14-day period of time and to determine appropriate doses for the 90-day study. The GLP study was conducted in compliance with OECD 407 (2008) [31] and FDA Redbook IV.C.3.a (2003) [32].

The test article was formulated just prior to administration in the vehicle (sunflower oil) and administered via gavage daily for 14 days at doses of 0, 1000, 2000, and 4000 mg/kg bw/day on the first day (day 0), and then the high dose was reduced to 3000 mg/kg bw/day on day 2 for humane reasons due to the mortality of one female animal in the 4000 mg/kg bw/day group. Some animals in the high-dose group (4000 mg/kg) were not dosed on day 1 (1 animal) or on day 2 (3 animals) due to toxic signs (for animal welfare reasons) and to avoid loss of further animals. Control animals were treated concurrently with vehicle only.

All animals were observed twice daily for morbidity and mortality. Dead animals were weighed and subjected to gross pathological examinations on the day of death and organs and tissues were processed and evaluated histologically. General cage-side observations for clinical signs were made twice during the acclimation period and once daily after administration of the test article. Detailed clinical observations were conducted once per day. An ophthalmologic examination was conducted during the acclimation period and prior to test termination on day 14.

Measurements of body weight were conducted twice during the acclimation period, on the first experimental day prior to treatment, and then twice weekly. Food consumption determinations coincided with body weight measurements.

After an overnight fast following final administration of the test article, blood samples were collected from the retro orbital venous plexus under Isoflurane CP® anesthesia (CP-Pharma Handelsgesellschaft mbH), after which the animals were euthanized by exsanguination from the abdominal aorta. Blood samples were analyzed for hematologic (white blood cells (WBC), red blood cells (RBC), hemoglobin (HGB), hematocrit (HCT), mean corpuscular volume (MCV), mean corpuscular hemoglobin (MCH), mean corpuscular hemoglobin concentration (MCHC), platelets (PLT), reticulocytes (RET), and WBC differential), blood coagulation (activated partial thromboplastin time (APTT) and prothrombin time (PT)), and clinical chemistry parameters (alanine transaminase (ALT), aspartate aminotransferase (AST), gamma-glutamyl transferase (GGT), alkaline phosphatase (ALP), total bilirubin (TBIL), creatinine (CREA), urea (UREA), glucose (GLUC), cholesterol (CHOL), bile acids (BAC), inorganic phosphorus (Pi), calcium (Ca^++^), sodium (Na^+^), potassium (K^+^), chloride (Cl^−^), albumin (ALB), total protein (TPROT), and albumin/globulin ratio (A/G)). Gross pathological examinations were conducted and selected absolute organ weights (liver, kidneys, testes, epididymides, uterus with fallopian tubes, thymus, spleen, brain, heart, adrenals, and ovaries) were measured and relative organ weights were calculated on all animals. Complete histopathological examinations were conducted on the preserved organs and tissues (adrenals, aorta, bone marrow of the femur, cerebrum, cerebellum, pons, medulla, eyes, mammary gland, gonads, heart, kidneys, large intestines, liver, lungs, submandibular and mesenteric lymph nodes, quadriceps muscle, esophagus, nasal turbinates, pancreas, pituitary, prostate, submandibular salivary glands, sciatic nerve, seminal vesicle, skin, small intestines, spinal cord at three levels, spleen, sternum, stomach, thymus, thyroid and parathyroid, trachea, and urinary bladder) of all animals of the control and high-dose groups.

Statistical analysis was done using SPSS PC+ software. The heterogeneity of variance between groups was checked by Bartlett's test. When no significant heterogeneity was detected, a one-way analysis was carried out. If the obtained result was positive, Duncan's multiple range test was used to assess the significance of intergroup differences. When significant heterogeneity was found, the normal distribution of data was examined by Kolmogorov-Smirnov test. In case of a nonnormal distribution, the nonparametric method of Kruskal-Wallis one-way ANOVA was used. If there was a positive result, the intergroup comparisons were performed using the Mann–Whitney *U* test. A* p* value of <0.05 was considered statistically significant.

#### 2.3.3. 90-Day Repeated Dose Oral Toxicity Study In Rats

A 90-day repeated dose oral toxicity study was conducted in Hsd.Han Wistar male and female rats in order to evaluate the possible health hazards likely to arise from repeated oral exposure to the test article during postweaning maturation and growth well into adulthood. The main study was followed by a 28-day recovery period in which two satellite groups (5 additional animals per sex per group in the control and high-dose groups) were observed in order to assess reversibility, persistence, or delayed occurrence of potential toxic effects. This GLP study was conducted in compliance with OECD 408 (1998) [33] and FDA Redbook IV.C.4.a (2003) [34].

The test article was formulated in the vehicle (sunflower oil) just prior to administration. The test article was administered via gavage at doses of 0, 100, 360, and 720 mg/kg bw/day at a dosing volume of 5 mL/kg bw. These doses were based on the 14-day study lowest observed adverse effect level (LOAEL) of 1000 mg/kg bw/day (the lowest dose group tested) with the aim of inducing moderately toxic effects in the middle- and high-dose groups (without causing mortality or suffering) and determining a no observed adverse effect level (NOAEL) in the low-dose group. Animals assigned to the satellite groups were treated identically up to day 90 and then observed without treatment for four weeks.

Healthy male (n = 50) and female (n = 50) Hsd.Han Wistar rats aged 42–52 days and weighing 155–186 g and 107–147 g, respectively, were acclimatized for seven days and randomly divided according to stratification by body weight into four groups (numbers include the satellite animals): 10/sex/group for the low- and mid-dose groups and 15/sex/group in the control and high-dose groups.

Animals were housed individually in type II polypropylene/polycarbonate cages in a room with 12-hour light-dark cycles, 10–15 air exchanges per hour via central air conditioner, at 22 ± 3°C, and a relative humidity of 30–70%.

All animals were observed twice daily for mortality. General cage-side observations for clinical signs were made twice during the acclimation period, once daily after administration of the test article during the treatment period and the recovery period. Detailed clinical observations were conducted on the day prior to the first treatment and once weekly thereafter during both the treatment and recovery periods. A functional observation battery (FOB) was performed during the final week of the study and included evaluation of sensory reactivity to stimuli, grip strength and motor activity, general physical condition, and behavior of the animals.

Individual body weights were recorded once during the acclimation period, on day 0 (prior to study start), twice weekly during weeks 1–4, and once weekly thereafter (weeks 5–13 and during the recovery period for satellite groups) and immediately prior to sacrifice (days 90, 91, and 118). Food consumption was determined weekly to coincide with body weight measurements and food efficiency was calculated once weekly. Ophthalmological examination was carried out on all animals prior to the experimental period and on control and high-dose group animals at the end of the treatment period.

After an overnight fast (approximately 16 hours) following final administration of the test article on days 90 and 91 and at the termination of the recovery period on day 118, blood samples were collected and animals euthanized as described in the 14-day study. Blood samples were analyzed for hematologic, blood coagulation, and clinical chemistry parameters (as listed in the 14-day study).

Gross pathological examinations and determinations of selected absolute and relative organ weights (compared to body weight and brain weight) (as listed for the 14-day study, plus thyroid/parathyroid) were conducted on all animals that were sacrificed on days 90 and 91, as well as those sacrificed at the end of the recovery period (day 118). Complete histopathological examinations were conducted on the preserved organs and tissues (as listed for the 14-day study) of all animals of the control and high-dose groups including animals of the recovery group. The histological examination of testes and epididymides covered the stages of spermatogenesis in the male gonads (spermatogonia, spermatocytes, spermatids, and spermatozoa) and histopathology of interstitial testicular cell structure. With regard to the prostate, seminal vesicle, and coagulating gland, the activity of secretion in the glandular tissue, the amount of secretions in the ducts, the average diameter of tubules, and the interstitial structures were evaluated.

The liver and adrenal glands were processed and evaluated histologically in all animals in the low- and mid-dose groups due to macroscopic findings at necropsy or organ weight changes. The kidneys of one male and one female animal at 100 mg/kg bw/day and one female animal at 360 mg/kg bw/day were also processed and evaluated histologically due to macroscopic findings at necropsy. Sperm analyses (qualitative and quantitative) were conducted on five animals from the control and high-dose groups from the treatment period (due to lack of any findings, these were not conducted in lower-dose groups or the recovery group animals). One testis per animal was used for enumeration of sperm, and sperms from the ductus deferens were collected for evaluation of sperm motility and sperm morphology. The examination was performed under a light microscope.

Qualitative examinations were performed immediately after euthanasia and exsanguination of animals. Approximately 0.5 cm of ductus deferens was placed in medium at 36°C for five minutes (allowing diffusion of sperm into the medium). One drop of this solution was then pipetted onto a glass slide and motile/immotile; normal and abnormal sperms were counted under a light microscope. For quantitative examination, testes were frozen at necropsy and enumeration was performed on the same animals used for qualitative examinations. After thawing, the testes were homogenized and dispensed in 10 mL of physiologic saline. The enumeration was performed by using Bürker chamber and sperm count was calculated using weight of testis and dilution volumes. Statistical analysis was performed as described in the 14-day study above.

## 3. Results

### 3.1. Bacterial Reverse Mutation Test

No substantial increases in revertant colony numbers were observed in any of the five tester strains following treatment with the test article in the presence or absence of metabolic activation (S9) at any concentration level (see Tables 1 and 2). Sporadic increases in revertant colony numbers compared to vehicle control were observed in both experiments, reflecting the biological variability of the applied test system; however, there was no tendency of dose related increases and mutation rates remained within the historical control data range.

### 3.2. In Vitro Mammalian Chromosomal Aberration Test

In the negative control group, the percentage of cells with structural aberrations was equal to or less than 5%, confirming the suitability of the V79 cell line used. The concurrent positive controls caused the expected biologically relevant increases of cells with structural chromosome aberrations as compared to current solvent and historical controls.

The test article did not induce an increase in the number of cells with aberrations or rates of polyploidy or endoreduplicated metaphases at concentrations ranging from 10 to 90 *μ*g/mL. There were no statistically significant differences between treatment and the solvent control groups, and no dose-response relationships were noted (see Table 3).

### 3.3. In Vivo Mouse Micronucleus Test

No mortality or gender specific effects were observed in the preliminary toxicity test; therefore, the micronucleus test was conducted at the doses described above in males only. In the main study, no mortality occurred. Adverse reactions to treatment were not observed in the positive controls, in negative controls, or in the 500 mg/kg bw group. A moderate decrease in activity, moderate restlessness, and slight/moderate irritability were observed in the 10 male mice treated with 1000 and 2000 mg/kg bw of the test article on the day of treatment. The mice did not exhibit any symptoms 24 and 48 hours after treatment. Because there was no mortality, bone marrow slides were not prepared on the two extra animals included in the high-dose group.

No significant differences were observed in frequency of MPCEs between the three dose groups compared to the negative control, and all results were within the laboratory's historical control range (see Table 4). Compared to the negative control group, the numbers of PCEs at 24- and 48-hour sampling times in the 500 and 1000 mg/kg bw groups were similar. In the 2000 mg/kg bw dose group, the number of PCEs was slightly decreased compared to the negative control group at the 24- and 48-hour sampling time points. The effect was not biologically significant but demonstrated exposure of the bone marrow to the test article. A large, statistically significant increase in MPCE frequency was observed in the positive control group compared to negative control. The cyclophosphamide-treated mice had MPCE counts that were slightly higher (61.40/2000 PCE) than historical controls (54.03/2000 PCE) but this deviation did not influence the quality or integrity of the study.

### 3.4. 14-Day Repeated Dose Oral Toxicity Study in Rats

One female animal died in the 4000 mg/kg bw/day group (highest dose group tested) on day 2, after which the dose of the test article for this group was decreased to 3000 mg/kg bw/day. Thereafter, three males and one female died in this group on days 4 (1 male), 5 (1 male), and 10 (1 male and 1 female). No mortality was observed in the 1000 and 2000 mg/kg bw/day groups. Clinical signs were noted in all test article treated groups and no clinical signs were noted in the control group. For example, restlessness and nuzzling up of the bedding material and salivation were noted in all animals of the 2000 and 3000/4000 mg/kg bw/day groups and one animal of each sex in the 1000 mg/kg bw/day group. Decreased activity and diarrhea were also noted in animals of the 2000 and 3000/4000 mg/kg bw/day groups and at least one animal of the high-dose group showed signs of tremor, prone positioning, narrow eye aperture, incoordination, or cold body temperature.

Mean body weight gain was statistically significantly decreased in all test groups compared to controls. Food consumption was statistically significantly reduced in animals of all test article groups throughout the study. Feed efficiency was also affected by treatment, with most animals experiencing a significant decrease; however, feed efficiency was not evaluated in some cases due to the body weight loss of the animals.

Hematological and clinical chemistry evaluations revealed various significant differences compared to controls, although many were marginal and not dose-dependent and/or fell well within historical control ranges (See Tables 5 and 6).

Macroscopic findings were detected at necropsy in animals that died prematurely at 3000/4000 mg/kg bw/day and included but were not limited to the following: dark red and enlarged liver, yellowish spots on the liver, smaller than normal spleen, seminal vesicles and prostate, dark red lung, dilated stomach, and fluid intestinal content. In surviving animals, the following were detected at necropsy: pale adrenal glands (all dose groups), dark brown liver (2000 and 3000/4000 mg/kg bw/day groups), smaller than normal spleen (one female in the 3000/4000 mg/kg bw/day group), smaller than normal thymus (2000 and 3000/4000 mg/kg bw/day groups), smaller than normal seminal vesicle and prostate (all dose groups), and undernourishment (2000 and 3000/4000 mg/kg bw/day groups). In two male animals at 2000 mg/kg bw/day, the testes were smaller than normal.

**Figure 1:**
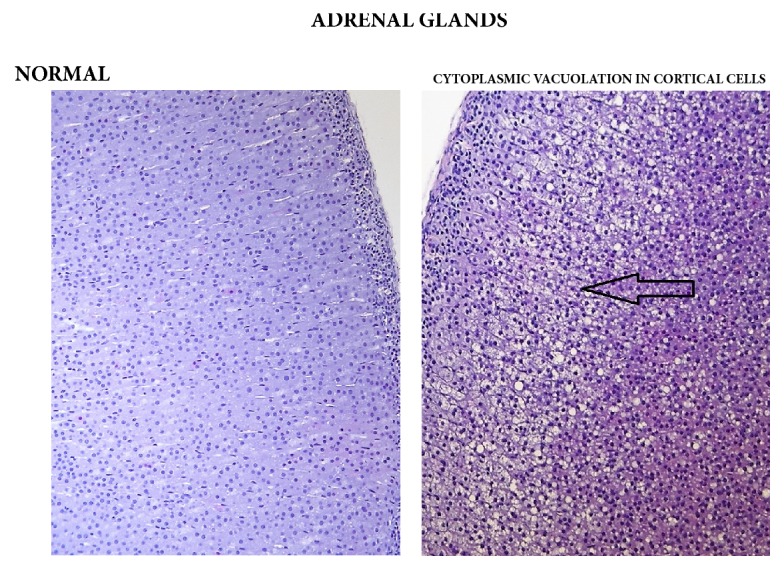
Adrenal cortex: comparison of normal (control) cortical cells to cells in a male from the 3000/4000 mg/kg bw/day group showing cytoplasmic vacuolation, 14-day study.

**Figure 2:**
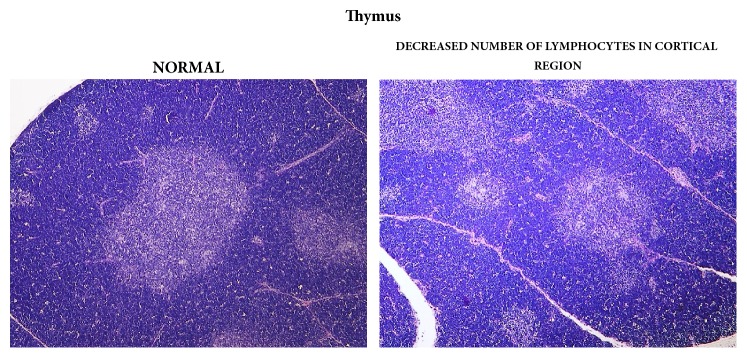
Thymus gland: comparison of normal (control) tissue to tissue in a male from the 3000/4000 mg/kg bw/day group showing decreased number of cortical lymphocytes, 14-day study.

**Figure 3:**
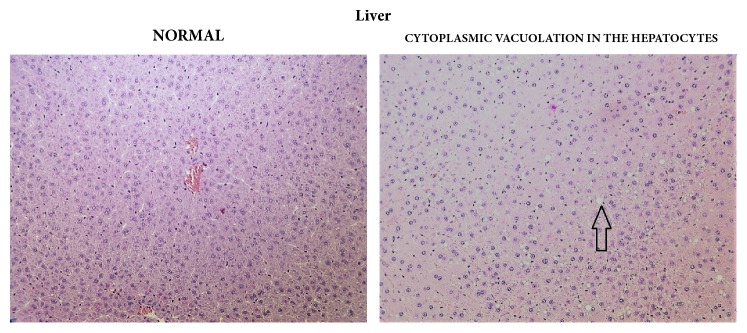
Liver: comparison of normal (control) tissue to hepatocytes of a male from the 3000/4000 mg/kg bw/day group showing cytoplasmic vacuolation, 14-day study.

**Figure 4:**
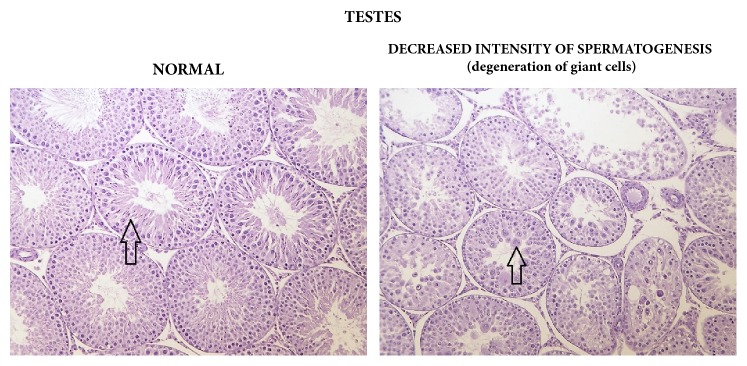
Testes: comparison of normal (control) tissue to tissue in a male from the 3000/4000 mg/kg bw/day group showing decreased intensity of spermatogenesis, 14-day study.

**Figure 5:**
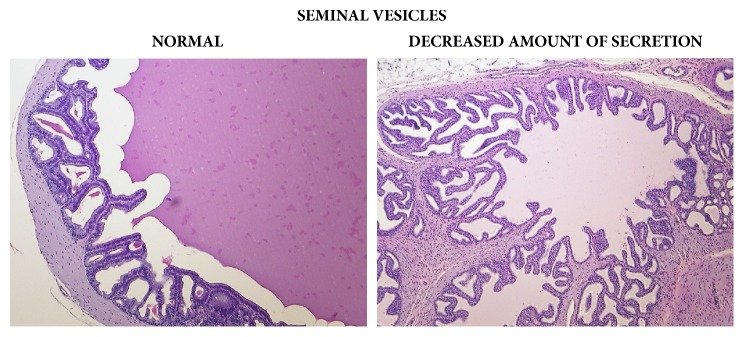
Seminal vesicles: comparison of normal (control) tissue to tissue from a male from the 3000/4000 mg/kg bw/day group showing decreased amount of secretion, 14-day study.

Statistically significant, apparently dose-related changes in the absolute and relative weights of liver, thymus, spleen, and adrenal glands at 1000, 2000, or 3000/4000 mg/kg bw/day were noted. Statistically significant absolute and relative changes in various other organ weights were noted in all dose groups. Histological examination of these organs revealed alveolar cytoplasmic vacuolation in the cortical zones of adrenal glands, cytoplasmic vacuolation of hepatocytes in the liver and of proximal convoluted tubules in the kidneys, accelerated involution of thymus, and lymphocyte depletion in the spleen (see Figures 1–3). With regard to the male reproductive system, decreased amounts of (Grade 1) or lack of (Grade 2) secretion in the seminal vesicles or prostate and decreased average diameter of the tubules were observed; additionally, a lack of mature spermatozoa and spermatids was observed in a proportion of seminiferous tubules, indicating decreased intensity of spermatogenesis (Grade 1, 10–20%; Grade 2, 20–50%; Grade 3, 50–60%) in the testes (see Figures 4 and 5). The presence of giant cells was observed.

### 3.5. 90-Day Repeated Dose Oral Toxicity Study In Rats

No deaths occurred in any dose groups throughout the main study period (0, 100, 360, and 720 mg/kg bw/day) or during the satellite groups recovery period (0 and 720 mg/kg bw/day (high dose)). No abnormal clinical signs were seen in either sex of the control group or in males of the 100 mg/kg bw/day group. In one female at 100 mg/kg bw/day, sanguineous fur around the eyes was detected between days 39 and 42. Clinical signs were observed in all animals in the 360 and 720 mg/kg bw/day groups. Nuzzling up the bedding material occurred in the 360 mg/kg bw/day group from day 20 or 21 up to the end of the treatment period. In the 720 mg/kg bw/day groups, nuzzling up the bedding material and restlessness were observed throughout the study. Salivation occurred in males (*n* = 7) and females (*n* = 4) of the 720 mg/kg bw/day shortly after administration of the test article during the first four weeks of the study. No further signs were found in detailed clinical observations in any dose group. No alterations in behavior or in reactions to various stimuli were noted in the FOB (data not shown). No clinical signs were observed in the satellite groups during the recovery period.

Significant decreases in body weight were detected in males in the 360 and 720 mg/kg bw/day groups and in females in the 720 mg/kg bw/day group as compared to controls (see Table 7). In the high-dose satellite group, these differences did not return to normal during the recovery period, although mean body weight gain was higher than controls in males from day 96 to the end of the recovery period with statistical significance between days 96–103 and days 110–117 (see Table 8). Body weight gain was not significantly affected in animals in the 100 mg/kg bw/day group, with the exception of a lower mean body weight gain in male animals between days 70 and 77, which did not correlate with a difference in mean body weight on day 77. However, similar to body weight, body weight gain was significantly lower with respect to the control group in males of the 360 mg/kg bw/day group and males and females in the 720 mg/kg bw/day group, although the mean body weight gain of females in the 720 mg/kg bw/day slightly exceeded control values between days 17 and 21.

Food consumption was significantly decreased compared to controls in males and females in the 360 and 720 mg/kg bw/day treatment groups from week 1 until the end of the treatment period, correlating with body weight differences (see Table 9). These differences also did not fully return to normal in the high-dose satellite group during the recovery period. Lower mean food consumption compared to controls was noted sporadically in males and females in the 100 mg/kg bw/day group. Statistically significant lower mean food consumption compared to controls was also noted in high-dose satellite animals in recovery week 1 (male) and in recovery weeks 1 and 4 (females). Slight, sporadic, yet statistically significant differences were noted in feed efficiency in all treatment groups (data not shown).

Ophthalmoscopic evaluation did not reveal any alterations to the eyes of animals in the control or 720 mg/kg bw/day group at the end of the treatment period (data not shown); therefore, no ophthalmoscopic examinations were conducted on satellite group animals during the recovery period. Several alterations in hematology and clinical chemistry parameters were noted during the 90-day period compared to the control group. Hematological differences were not found in the high-dose group at the conclusion of the recovery period; however, some differences in clinical chemistry were still present during this timeframe (see Tables 10 and 11).

Upon necropsy, enlarged and pale adrenal glands were noted in male (5/10) and female (7/10) animals in the 720 mg/kg bw/day group. Mottled surface of the kidneys was also noted in one male (1/10) in the 100 mg/kg bw/day group and in two females (1/10 in the 100 mg/kg bw/day group and 1/10 in the 360 mg/kg bw/day group). Slight or moderate hydrometra of the uterus was observed in some females (4/10 in the control group, 2/10 in the 100 mg/kg bw/day group, and 2/10 in the 720 mg/kg bw/day group). At the end of the recovery period, no macroscopic findings were noted in satellite group males. In satellite group females, slight or moderate hydrometra was observed in both control and high-dose groups (3/5 and 1/5, resp.).

Statistically significant differences with respect to control were noted in several absolute and relative organ weight measures in the test article groups during the main study. Several differences in organ weights in high-dose males and females were also identified during the recovery period (see Tables 12–14).

Total sperm count, sperm morphology, and percentage of motile and immotile sperm cells were similar in the control and 720 mg/kg bw/day groups at the end of the treatment period. Therefore, no sperm examinations were conducted during the recovery period.

**Figure 6:**
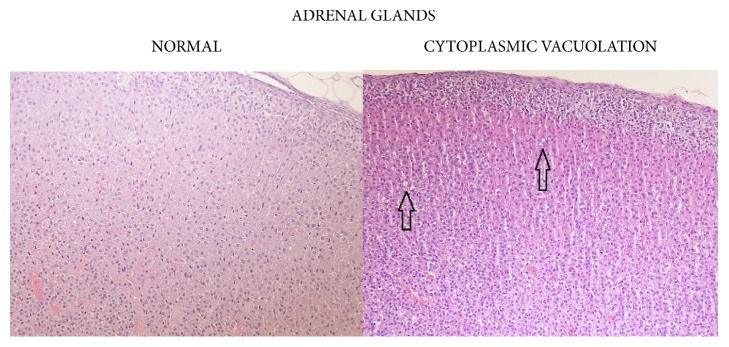
Adrenal cortex: comparison of normal (control) cortical cells to cells in an animal from the 720 mg/kg bw/day group showing cytoplasmic vacuolation, 90-day study.

Histopathological examination revealed mild-to-moderate diffuse cytoplasmic vacuolation of the cortical cells of the adrenal glands (involving the zona fasciculata and zona reticularis) in male (6/10) and female (8/10) animals of the 720 mg/kg bw/day group, although these lesions were not detected in satellite groups at the end of the recovery period. A list of histopathological findings can be found in Table 15 and photos can be found in Figure 6.

## 4. Discussion and Conclusions

The current studies were undertaken to better understand the toxicological profile of this hemp extract rich in CBD in OECD compliant in vitro and animal studies. The bacterial reverse mutation, in vitro mammalian chromosomal aberration, and in vivo mouse micronucleus tests met their respective validity and sensitivity criteria and were unequivocally negative under the conditions of their respective study.

In the 14-day repeated dose oral toxicity study, a NOAEL in Hsd.Han Wistar rats could not be established because of test article related adverse toxicological effects and histopathological findings. Thus, lower dose levels were chosen for the subchronic study.

In the 90-day study, while nuzzling up bedding material, restlessness, and salivation were noted in both sexes of the 360 and 720 mg/kg bw/day groups during the treatment period, these behaviors were not observed during the recovery period; therefore, they were considered a reversible effect of the test article. Slight, statistically significant differences in body weight and food consumption in male and female rats in the 100 mg/kg bw/day group were not considered to be toxicologically relevant due to the low degree of change and their sporadic occurrence. In contrast, the reduced body weight gain noted in the 360 (males only) and 720 (both sexes) mg/kg bw/day groups was considered test article related. The reduced food consumption observed in both sexes in the 360 and 720 mg/kg bw/day groups may also explain the decreased body weight gain. For high-dose satellite group males and females, decreased food consumption and body weights persisted throughout the recovery period.

The statistically significant differences noted for hematological, blood coagulation, and clinical chemistry parameters in all test article-administered groups were not considered toxicologically relevant due to a small degree of change, lack of dose-dependence, and/or the fact that the values remained within historical control ranges. Statistically significantly reduced RET values in female animals in the 360 and 720 mg/kg bw/day groups fell well within historical control values and were not accompanied by any signs of bone marrow lesions; thus, the reductions were not considered clinically relevant. Statistically significantly elevated levels of GGT in male and female animals in the 360 and 720 mg/kg bw/day groups were associated with liver weight changes but were without any related histopathological changes. Therefore, the elevation of GGT was considered to be an adaptive response to the altered demand due to the presence of the test article, that is, the physiological response of an organism in order to maintain normal function [35, 36]. While GGT remained statistically significantly higher in the high-dose satellite group males and females during the recovery period, the values returned to historical control ranges; therefore, the change in GGT appeared to be reversible. CREA in high-dose satellite group females became statistically significantly lower compared to satellite controls during the recovery period. This change in significance could be attributed to the increase in the satellite control CREA at the end of recovery period (34.44 ± 2.50) compared to the end of the 90-day treatment period (31.81 ± 2.69) rather than the change in the high-dose satellite group, which increased from 30.37 ± 2.35 at the end of the 90-day treatment period to 30.72 ± 2.12 at the end of the recovery period (see Table 7). The sperm examinations did not reveal any test article related influence at 720 mg/kg bw/day.

The changes noted in fasted body weight and in the absolute and/or relative weights of the liver and adrenal glands in both male and female animals of the 360 and 720 mg/kg bw/day groups were considered to be test article related due to the associated changes in GGT of males and females in both of those groups and the pale and enlarged adrenal glands noted in macroscopic observations (in males and females of the 720 mg/kg bw/day group only). In the high-dose groups, histopathological examination revealed that pale adrenal glands were accompanied by increased diffuse cytoplasmic vacuolation of the cortical cells of the adrenal glands in male and female rats. The cytoplasmic vacuolation of the cortical cells is usually associated with increased cellular activity and may be secondary to xenobiotic treatment or due to stress [37, 38]. These lesions were not found in the treated rats at the end of the recovery period and therefore were considered to be reversible. Histopathological examinations also revealed alveolar emphysema, which was considered to be due to the hypoxia, dyspnea, and circulatory disturbance commonly developed during exsanguination and alveolar histiocytosis, which is a common incidental finding in aging rats [39]. Dilatation of uterine horns was considered a neurohormonal phenomenon associated with the proestrus phase of the inner reproductive organs [40]. Renal findings in single animals described above (cyst, mineral deposits, and focal fibrosis) were judged to be individual disorders without toxicological significance [41, 42]. No toxicologically relevant, test article related changes were observed in male or female animals given 100 mg/kg bw/day or in female animals given 360 mg/kg bw/day for 90 days.

In conclusion, the test article was considered nonmutagenic, nonclastogenic, and nongenotoxic in the current bacterial reverse mutation, in vitro mammalian chromosomal aberration, and in vivo mouse micronucleus tests, respectively. The NOAEL for the test article in this 90-day study was considered to be 100 mg/kg bw/day for male and 360 mg/kg bw/day for female Hsd.Han Wistar rats. The toxicological assessment that is reported herein is the first known of its kind since the 1981 Rosenkrantz et al.'s publication with respect to published toxicology data on CBD, hemp, or hemp extracts [15]. Given the broad physiological actions of CBD and other hemp-derived phytocannabinoids, this battery of OECD-compliant toxicological studies is a salient contribution to the literature, providing a more extensive assessment of this supercritical CO_2_ extract of the aerial parts of the hemp (*C. sativa*) plant.

## Figures and Tables

**Table 1:** Summary table of the results of the initial mutation test.

Initial mutation test (plate incorporation test)*∗*																				

Concentrations (*μ*g/plate)	*Salmonella typhimurium* tester strains																*Escherichia coli*			
	TA 98				TA 100				TA 1535				TA 1537				WP2uvrA			
	-S9		+S9		-S9		+S9		-S9		+S9		-S9		+S9		-S9		+S9	

Mean values of revertants per plate and mutation rate (MR)	Mean	MR	Mean	MR	Mean	MR	Mean	MR	Mean	MR	Mean	MR	Mean	MR	Mean	MR	Mean	MR	Mean	MR

Untreated control	13.7	0.89	19.3	1.09	84.0	1.02	105.0	1.12	8.7	1.18	11.7	1.03	12.7	0.93	11.3	0.89	13.3	0.95	21.7	1.16
DMSO control	15.3	1.00	17.7	1.00	82.0	1.00	93.7	1.00	7.3	1.00	11.3	1.00	13.7	1.00	12.7	1.00	14.0	1.00	18.7	1.00
Ultrapure Water control	–	–	–	–	83.3	1.00	–	–	8.3	1.00	–	–	–	–	–	–	14.0	1.00	–	–

5000	11.0	0.72	14.0	0.79	54.0	0.66	78.0	0.83	3.7	0.50	4.7	0.41	4.7	0.34	2.0	0.16	13.0	0.93	18.0	0.96
1600	13.3	0.87	18.0	1.02	56.0	0.68	70.3	0.75	9.3	1.27	8.0	0.71	12.7	0.93	9.7	0.76	10.7	0.76	18.0	0.96
500	12.3	0.80	22.0	1.25	60.7	0.74	86.3	0.92	8.7	1.18	10.0	0.88	10.0	0.73	12.0	0.95	11.3	0.81	19.3	1.04
160	11.0	0.72	23.0	1.30	89.3	1.09	92.3	0.99	8.0	1.09	11.3	1.00	11.7	0.85	12.7	1.00	14.3	1.02	19.7	1.05
50	15.3	1.00	24.0	1.36	98.3	1.20	87.0	0.93	7.3	1.00	9.7	0.85	12.0	0.88	13.7	1.08	11.0	0.79	16.7	0.89
16	15.3	1.00	20.0	1.13	86.0	1.05	91.7	0.98	9.0	1.23	9.0	0.79	12.0	0.88	12.7	1.00	14.0	1.00	17.7	0.95
5	13.3	0.87	17.0	0.96	84.3	1.03	101.0	1.08	9.0	1.23	12.0	1.06	13.3	0.98	12.3	0.97	14.0	1.00	18.3	0.98

NPD (4)	208.0	13.57	–	–	–	–	–	–	–	–	–	–	–	–	–	–	–	–	–	–
SAZ (2)	–	–	–	–	1008.0	12.10	–	–	1066.7	128.00	–	–	–	–	–	–	–	–	–	–
9AA (50)	–	–	–	–	–	–	–	–	–	–	–	–	574.0	42.00	–	–	–	–	–	–
MMS (2 *μ*L)	–	–	–	–	–	–	–	–	–	–	–	–	–	–	–	–	422.7	30.19	–	–
2AA (2)	–	–	1469.3	83.17	–	–	2506.7	26.76	–	–	105.0	9.26	–	–	136.7	10.79	–	–	–	–
2AA (50)	–	–	–	–	–	–	–	–	–	–	–	–	–	–	–	–	–	–	209.0	11.20

**∗**DMSO was used as the vehicle for the test article and positive control substances: NPD, 9AA, and 2AA. Ultrapure water was used as the vehicle for SAZ and MMS. The mutation rate of the test item and the untreated control is given referring to the DMSO.

**Table 2:** Summary table of the results of the confirmatory mutation test.

Confirmatory mutation test (preincubation test)*∗*																				

Concentrations (*μ*g/plate)	*Salmonella typhimurium* tester strains																*Escherichia coli*			
	TA 98				TA 100				TA 1535				TA 1537				WP2uvrA			
	-S9		+S9		-S9		+S9		-S9		+S9		-S9		+S9		-S9		+S9	

Mean values of revertants per plate and mutation rate (MR)	Mean	MR	Mean	MR	Mean	MR	Mean	MR	Mean	MR	Mean	MR	Mean	MR	Mean	MR	Mean	MR	Mean	MR

Untreated control	14.7	0.96	22.7	1.33	102.0	1.02	126.3	1.10	8.7	0.93	8.0	0.83	9.3	1.27	9.0	0.84	13.3	0.77	19.3	1.16
DMSO control	15.3	1.00	17.0	1.00	100.0	1.00	114.3	1.00	9.3	1.00	9.7	1.00	7.3	1.00	10.7	1.00	17.3	1.00	16.7	1.00
Ultrapure water control	–	–	–	–	86.7	1.00	–	–	13.0	1.00	–	–	–	–	–	–	17.7	1.00	–	–

5000	5.0	0.33	16.7	0.98	44.3	0.44	61.3	0.54	0.3	0.04	9.0	0.93	0.7	0.09	4.7	0.44	12.7	0.73	16.3	0.98
1600	9.0	0.59	23.3	1.37	46.7	0.47	71.3	0.62	2.7	0.29	8.3	0.86	2.0	0.27	8.7	0.81	16.3	0.94	17.7	1.06
500	8.3	0.54	18.0	1.06	45.7	0.46	69.7	0.61	6.0	0.64	9.3	0.97	0.7	0.09	8.3	0.78	14.3	0.83	15.3	0.92
160	12.3	0.80	26.7	1.57	80.3	0.80	91.0	0.80	8.3	0.89	10.0	1.03	4.3	0.59	9.3	0.88	10.7	0.62	20.3	1.22
50	12.3	0.80	26.3	1.55	94.7	0.95	100.7	0.88	10.0	1.07	10.0	1.03	7.7	1.05	10.3	0.97	16.0	0.92	18.0	1.08
16	14.0	0.91	29.0	1.71	94.7	0.95	101.0	0.88	9.3	1.00	8.7	0.90	7.7	1.05	11.7	1.09	12.3	0.71	19.3	1.16
5	14.3	0.93	23.0	1.35	94.7	0.95	115.7	1.01	10.3	1.11	13.0	1.34	7.3	1.00	11.3	1.06	16.0	0.92	18.3	1.10

NPD (4)	310.0	20.22	–	–	–	–	–	–	–	–	–	–	–	–	–	–	–	–	–	–
SAZ (2)	–	–	–	–	1389.3	16.03	–	–	3104.0	238.77	–	–	–	–	–	–	–	–	–	–
9AA (50)	–	–	–	–	–	–	–	–	–	–	–	–	586.0	79.91	–	–	–	–	–	–
MMS (2 *μ*L)	–	–	–	–	–	–	–	–	–	–	–	–	–	–	–	–	898.7	50.87	–	–
2AA (2)	–	–	989.3	58.20	–	–	1752.0	15.32	–	–	113.3	11.72	–	–	133.7	12.53	–	–	–	–
2AA (50)	–	–	–	–	–	–	–	–	–	–	–	–	–	–	–	–	–	–	181.3	10.88

**∗**DMSO was applied as vehicle of the test article and positive control substances: NPD, 9AA, and 2AA; and the ultrapure water was applied as vehicle for the SAZ and MMS. The mutation rate of the test item and the untreated control is given referring to the DMSO.

**Table 3:** Summary of chromosomal aberration test results.

**Concentration**	S9 mix	Treatment	Harvest	Mean aberrant cells (per 200 cells)		Number of aberrations	
*μ*g/mL		Time (h)	Time(h)	Incl. gaps	Excl. gaps	Incl. gaps	Excl. gaps
	**Experiment A** ^**1**^						

Test Article							
10	–	3	20	7	4	7	4
20	–	3	20	6	3	8	3
30	–	3	20	7	4	8	4
Vehicle control	–	3	20	6	3	6	3
Positive control	–	3	20	60∗∗	48∗∗	100∗∗	63∗∗
Hist. veh. control^4^	–	3	20	0.35–11.99	0.00–7.30	n/a	n/a

Test Article							
50	+	3	20	8	5	9	5
70	+	3	20	7	3	7	3
90	+	3	20	9	5	10	5
Vehicle control	+	3	20	7	4	7	3
Positive control	+	3	20	69∗∗	58∗∗	130∗∗	85∗∗
Hist. veh. control^4^	+	3	20	0.00–15.19	0.53–5.47	n/a	n/a

	**Experiment B** ^**2**^						

Test Article							
1.25	–	20	20	6	2	6	2
2.5	–	20	20	6	3	6	3
5	–	20	20	8	3	8	3
Vehicle control	–	20	20	6	3	7	3
Positive control	–	20	20	55∗∗	48∗∗	112∗∗	60∗∗
Hist. veh. control^4^	–	20	20	2.58–10.42	1.39–4.94	n/a	n/a

	**Experiment B** ^**3**^						

Test Article							
1.25	–	20	28	8	4	8	4
2.5	–	20	28	7	4	8	4
5	–	20	28	8	4	8	4
Vehicle control	–	20	28	6	3	6	3
Positive control	–	20	28	67∗∗	62∗∗	146∗∗	84∗∗
Hist. veh. control^4^	–	20	28	2.70–11.30	3.00–3.00	n/a	n/a

Test Article							
50	+	3	28	9	4	10	5
70	+	3	28	9	4	10	4
90	+	3	28	7	3	8	4
Vehicle control	+	3	28	7	4	7	4
Positive control	+	3	28	63∗∗	54∗∗	140∗∗	105∗∗
Hist. veh. control^4^	+	3	28	4.73–8.27	0.00–6.97	n/a	n/a

^1^Positive controls: (–S9): ethyl methanesulfonate (1.0 *μ*L/mL); (+S9): cyclophosphamide (5.0 *μ*g/mL).

^2^Positive control: (–S9): ethyl methanesulfonate (0.4 *μ*L/mL).

^3^Positive controls: (–S9) ethyl methanesulfonate (0.4 *μ*L/mL); (+S9): cyclophosphamide (5.0 *μ*g/mL).

^4^Numbers reported are the 95% confidence interval.

*∗∗*p < 0.01 to the concurrent vehicle control and to the historical vehicle control.

n/a, not available; veh., vehicle; Hist., historical; Incl., including; Excl., excluding.

**Table 4:** Summary table of the results for the mouse micronucleus test.

Groups (mg/kg bw)	Sampling time (hour)	Total number of PCEs analyzed	MPCE (per 4000 PCE)		PCE/NCE	
			Mean	±SD	Mean	±SD
Negative control 0	24	20000	4.40	1.14	1.13	0.04
500	24	20000	4.60	0.55	1.09	0.12
1000	24	20000	4.60	1.14	1.06	0.08
2000	24	20000	5.20	0.84	0.91	0.03
Positive control 60	24	20000	122.80∗∗	6.02	0.46	0.10

Negative control 0	48	20000	4.40	0.89	1.12	0.12
2000	48	20000	5.20	1.30	0.94	0.10

Positive control: cyclophosphamide.

Negative control: 1% aqueous methylcellulose.

∗∗: p < 0.01 to the concurrent negative control and to the historical control.

**Table 5:** Summary of selected† hematological findings in the 14-day repeated oral toxicity study.

Group	WBC	NEU	LYM	MONO	EOS	RBC	HCT	MCV	MCH	MCHC	PLT	RET
(mg/kg bw/day)	×10^9^/L	%	%	%	%	×10^12^/L	L/L	fL	pg	g/L	×10^9^/L	%
Male (main groups, *n* = 5 each unless otherwise noted)												
Control	7.56 ± 1.03	14.90 ± 1.96	82.56 ± 2.13	1.78 ± 0.78	0.74 ± 0.34	8.17 ± 0.71	0.44 ± 0.04	53.70 ± 2.47	19.06 ± 0.78	355.20 ± 5.93	773.40 ± 66.89	4.74 ± 0.58
1000	8.20 ± 1.54	15.60 ± 3.16	82.20 ± 3.09	1.66 ± 0.51	0.46 ± 0.15	8.59 ± 0.54	0.45 ± 0.02	52.98 ± 2.12	19.02 ± 0.74	360.20 ± 2.59	868.00 ± 47.65	2.96 ± 0.39∗∗
2000	7.03 ± 1.40	9.92 ± 4.69*∗*	88.90 ± 5.21*∗*	1.06 ± 0.39	0.12 ± 0.16∗∗	8.92 ± 0.72	0.45 ± 0.01	50.62 ± 3.30	18.28 ± 0.87	361.80 ± 7.60	901.80 ± 93.29*∗*	2.51 ± 1.30∗∗
3000†† (n = 2)	14.44 ± 2.59∗∗	13.35 ± 1.91	82.90 ± 2.97	3.35 ± 1.20*∗*	0.30 ± 0.14*∗*	9.17 ± 0.06	0.44 ± 0.02	48.30 ± 1.84*∗*	18.40 ± 0.28	380.50 ± 9.19∗∗	1009.50 ± 44.55∗∗	1.49 ± 1.22∗∗
Historical range*♮*	6.23–13.73	6.8–28.2	68.5–91.6	0.8–3.7	0.1–2.2	6.6–9.5	0.37–0.50	48.7–60.8	17.9–21.1	348–377	631–1185	3.35–6.73

Female (main groups, *n* = 5 each unless otherwise noted)												
Control	6.21 ± 1.16	10.46 ± 2.87	88.02 ± 3.06	1.02 ± 0.33	0.50 ± 0.22	8.10 ± 0.22	0.44 ± 0.01	54.14 ± 0.90	19.40 ± 0.33	358.60 ± 3.21	913.40 ± 37.32	4.62 ± 0.82
1000	7.24 ± 3.22	10.36 ± 3.69	88.06 ± 4.01	0.94 ± 0.30	0.62 ± 0.22	8.61 ± 0.34*∗*	0.46 ± 0.01	52.98 ± 2.57	18.90 ± 0.75	356.80 ± 5.22	887.00 ± 109.73	3.59 ± 0.53
2000	7.31 ± 2.77	13.26 ± 8.36	85.28 ± 8.22	1.12 ± 0.41	0.30 ± 0.10	8.76 ± 0.28*∗*	0.45 ± 0.01	51.10 ± 1.08*∗*	18.38 ± 0.36∗∗	359.60 ± 7.16	914.60 ± 87.37	3.44 ± 0.92
3000†† (n = 2)	4.13 ± 1.49	26.35 ± 20.86	72.20 ± 21.78	1.45 ± 0.92	0.00 ± 0.00∗∗	8.26 ± 0.63	0.42 ± 0.02*∗*	50.35 ± 1.20*∗*	17.95 ± 0.07∗∗	357.00 ± 9.90	1551.00 ± 599.63	5.58 ± 5.82
Historical range*♮*	4.07–10.67	5.5–24.2	71.7–92.5	0.7–3.5	0.2–2.7	7.8–9.1	0.41–0.47	48.1–57.8	17.7–20.5	353–374	635–1118	2.56–6.97

Data represents the mean values and the standard deviation.

Dose was reduced from 4000 to 3000 mg/kg bw/d on day 2.

†: only parameters with statistically significant findings are shown in table.

††: dose was reduced from 4000 to 3000 mg/kg bw/d on day 2.

*∗P* < 0.05 and ∗∗*P* < 0.01, significance determined with Duncan's multiple range test or with Mann–Whitney *U* test versus control.

*♮*: minimum and maximum levels reported as the range of historical control values.

**Table 6:** Summary of selected† clinical chemistry findings in the 14-day repeated oral toxicity study.

Group	ALT	GGT	ALP	TBIL	CREA	Pi	Na^+^	Cl^−^	ALB
(mg/kg bw/day)	U/L	U/L	U/L	*μ*mol/L	*μ*mol/L	mmol/L	mmol/L	mmol/L	mmol/L
Male (main groups, *n* = 5 each, except 3000 mg/kg bw/day group, *n* = 2)									
Control	49.74 ± 6.56	0.30 ± 0.46	233.00 ± 51.06	2.25 ± 0.43	21.66 ± 1.45	2.69 ± 0.23	142.80 ± 1.48	102.70 ± 0.58	34.14 ± 0.84
1000	58.00 ± 8.98	1.92 ± 0.68∗∗	182.60 ± 31.83	1.79 ± 0.21	20.96 ± 1.38	2.34 ± 0.36	142.40 ± 1.14	102.88 ± 0.64	33.86 ± 0.67
2000	79.10 ± 30.74	11.94 ± 4.60∗∗	312.80 ± 54.34*∗*	4.09 ± 1.15∗∗	20.78 ± 1.48	2.55 ± 0.24	142.40 ± 1.14	102.70 ± 1.06	33.80 ± 1.49
3000††	140.70 ± 83.72	29.80 ± 14.00	452.00 ± 84.85∗∗	16.27 ± 11.79	18.70 ± 0.00	2.43 ± 0.01	140.50 ± 2.12	102.05 ± 0.92	31.45 ± 3.89
Historical range*♮*	26.5–87.9	0.0–1.2	111–316	1.01–2.69	17.2–31.1	2.20–3.10	136.0–145.0	99.7–106.2	31.8–36.8

Female (main groups, *n* = 5 each, except 3000 mg/kg bw/d group, *n* = 3)									
Control	44.82 ± 8.55	1.72 ± 0.26	119.60 ± 15.44	1.96 ± 0.23	21.30 ± 1.74	2.38 ± 0.23	142.60 ± 0.89	103.66 ± 0.56	34.64 ± 0.47
1000	67.82 ± 23.86	5.32 ± 2.03∗∗	135.00 ± 32.14	2.01 ± 0.20	20.50 ± 2.28	2.32 ± 0.25	142.40 ± 0.55	104.14 ± 1.92	33.92 ± 0.67
2000	87.92 ± 16.57∗∗	16.76 ± 4.09∗∗	234.00 ± 54.18∗∗	4.34 ± 2.25∗∗	17.58 ± 1.38*∗*	2.57 ± 0.23	141.20 ± 2.95	102.74 ± 2.64	33.76 ± 0.42*∗*
3000††	76.93 ± 18.50*∗*	41.80 ± 30.45*∗*	228.67 ± 78.49∗∗	6.16 ± 2.11*∗*	13.93 ± 3.46∗∗	2.98 ± 0.44*∗*	144.67 ± 1.15*∗*	107.47 ± 2.65*∗*	32.00 ± 2.69
Historical range*♮*	21.3–74.2	0.1–3.3	53–194	0.89–2.26	19.4–37.3	1.71–3.13	137.0–145.0	101.6–107.9	32.4–38.5

Data represent the mean values and the standard deviation.

†: only parameters with statistically significant findings are shown in table.

††: dose was reduced from 4000 to 3000 mg/kg bw/d on day 2.

*∗P* < 0.05 and ∗∗*P* < 0.01, significance determined with Duncan's multiple range test or with Mann–Whitney *U* test versus control.

*♮*: minimum and maximum levels reported as the range of historical control values.

**Table:** (a) Summary of body weights in the 90-day repeated oral toxicity study

Group (mg/kg bw/day)	Body weight (g) on days																	
	0	3	7	10	14	17	21	24	28	35	42	49	56	63	70	77	84	89
Males																		
Control	170.9 ± 10.1	190.3 ± 10.2	214.7 ± 11.0	232.9 ±10.6	252.1 ± 12.2	267.1 ± 12.8	281.5 ± 13.8	292.7 ± 14.9	305.0 ± 15.5	325.7 ± 16.8	338.7 ± 17.5	351.9 ± 20.1	360.7 ± 20.8	369.5 ± 21.3	379.7 ± 22.7	385.8 ± 23.8	397.1 ± 26.0	397.3 ± 25.4
100	172.0 ±8.4	191.3 ± 10.9	214.9 ± 13.3	231.8 ± 16.9	251.3 ± 20.8	266.5 ± 23.2	280.7 ± 26.2	293.0 ± 29.1	304.6 ± 32.3	325.1 ± 36.0	337.5 ± 37.7	349.0 ± 39.4	354.8 ± 39.3	361.8 ± 41.5	370.2 ± 42.9	371.8 ± 42.7	382.5 ± 43.1	386.1 ± 45.1
360	171.1 ± 8.6	180.7 ± 7.6*∗*	199.4 ± 8.9∗∗	215.1 ± 9.2∗∗	230.9 ± 9.3∗∗	244.8 ± 8.9∗∗	257.6 ± 9.8∗∗	268.3 ± 10.9∗∗	278.8 ± 11.3∗∗	296.3 ± 12.8∗∗	308.7 ± 14.8∗∗	318.3 ± 15.5∗∗	323.2 ± 17.6∗∗	332.0 ± 19.4∗∗	341.5 ± 19.8∗∗	344.5 ± 20.5∗∗	356.7 ± 22.7∗∗	358.4 ± 19.5∗∗
720	171.3 ± 7.1	175.2 ± 6.5∗∗	193.1 ± 6.2∗∗	205.5 ± 7.5∗∗	222.3 ±8.7∗∗	233.3 ± 9.6∗∗	247.0 ± 11.7∗∗	256.4 ± 12.5∗∗	266.9 ± 12.7∗∗	281.9 ± 12.3∗∗	294.8 ± 12.6∗∗	303.5 ± 12.4∗∗	311.3 ± 12.8∗∗	317.6 ± 14.5∗∗	326.0 ± 14.5∗∗	327.1 ± 14.8∗∗	339.7 ± 16.2∗∗	341.0 ± 17.9∗∗

Females																		
Control	124.3 ± 10.4	135.1 ± 10.8	147.1 ± 11.1	155.7 ± 10.1	164.3 ± 10.3	173.1 ± 10.0	179.8 ± 10.8	186.9 ± 10.8	193.3 ± 11.5	205.3 ± 12.7	213.5 ± 13.0	221.1 ± 14.6	224.9 ± 12.7	231.2 ± 14.6	236.0 ± 15.2	236.5 ± 15.9	245.1 ± 15.6	244.5 ± 17.0
100	125.7 ± 10.6	134.8 ± 10.0	146.0 ± 10.2	153.7 ± 11.4	162.8 ± 10.9	171.2 ± 9.9	178.5 ± 13.0	187.0 ± 13.0	193.6 ± 12.9	204.3 ± 13.7	210.3 ± 13.0	218.1 ± 14.9	221.8 ± 13.5	226.7 ± 14.6	232.4 ± 14.7	230.9 ± 16.0	241.8 ± 16.4	242.1 ± 17.1
360	122.8 ± 10.8	128.1 ± 10.7	138.9 ± 10.7	146.6 ± 10.8	156.5 ± 10.7	165.8 ± 11.5	173.3 ± 12.0	180.7 ± 11.9	186.4 ± 12.5	200.2 ± 13.1	206.8 ± 12.3	211.5 ± 13.5	216.3 ± 14.1	221.2 ± 13.3	224.3 ± 14.2	225.0 ± 13.2	231.7 ± 12.7*∗*	233.0 ± 11.0
720	123.7 ± 10.0	124.5 ± 8.6*∗*	132.9 ± 10.5∗∗	141.1 ± 10.3∗∗	151.3 ± 11.8∗∗	160.1 ± 10.4∗∗	169.1 ± 10.4*∗*	175.5 ± 10.1*∗*	182.5 ± 9.6*∗*	191.0 ± 10.6∗∗	198.4 ± 10.4∗∗	203.3 ± 10.8∗∗	206.4 ± 11.3∗∗	211.5 ± 10.5∗∗	214.2 ± 11.1∗∗	212.6 ± 11.8∗∗	219.1 ± 10.8∗∗	220.9 ± 11.1∗∗

*n* = 15 in control and 720 mg/kg bw/day groups; in all other groups *n* = 10.

*∗*p < 0.05, ∗∗p < 0.01, statistical significances were determined with Duncan's multiple range test or with Mann–Whitney *U* test versus control.

**Table:** (b) Summary of body weight in the recovery period

Group (mg/kg bw/day)	Body weight (g) on days			
	96	103	110	117
Males				
Control	422.0 ± 14.7	431.4 ± 17.6	440.6 ± 16.8	445.6 ± 16.4
720	344.6 ± 11.7∗∗	360.4 ± 14.0∗∗	373.2 ± 17.5∗∗	384.6 ± 22.1∗∗

Females				
Control	244.6 ± 9.1	247.6 ± 10.4	252.6 ± 10.0	254.8 ± 10.1
720	215.6 ± 9.8∗∗	218.0 ± 10.6∗∗	220.4 ± 14.9∗∗	226.8 ± 9.5∗∗

*n* = 5 for all groups.

*∗*p < 0.05, ∗∗p < 0.01, statistical significances were determined with Duncan's multiple range test or with Mann–Whitney *U* test versus control.

**Table:** (a) Summary of mean body weight gain in the 90-day repeated oral toxicity study

Group (mg/kg bw/day)	Body weight gain (g) between days																	
	0-3	3-7	7-10	10-14	14-17	17-21	21-24	24-28	28-35	35-42	42-49	49-56	56-63	63-70	70-77	77-84	84-89	0–89
Males																		
Control	19.5 ± 2.3	24.3 ± 3.0	18.3 ± 1.4	19.2 ± 3.7	14.9 ± 2.7	14.4 ± 3.0	11.3 ± 2.8	12.3 ± 2.8	20.7 ± 2.7	12.9 ± 2.9	13.3 ± 3.8	8.7 ± 3.5	8.8 ± 2.8	10.2 ± 2.9	6.1 ± 3.5	11.3 ± 3.6	0.2 ± 2.3	226.5 ± 24.5
100	19.3 ± 3.2	23.6 ± 4.3	16.9 ± 4.4	19.5 ± 6.1	15.2 ± 3.9	14.2 ± 4.6	12.3 ± 3.2	11.6 ± 3.3	20.5 ± 5.3	12.4 ± 2.5	11.5 ± 4.9	5.8 ± 2.9	7.0 ± 5.6	8.4 ± 2.6	1.6 ± 3.7∗∗	10.7 ± 2.8	3.6 ± 3.4	214.1 ± 40.6
360	9.6 ± 4.8∗∗	18.7 ± 2.9∗∗	15.7 ± 3.3*∗*	15.8 ± 2.7	13.9 ± 3.0	12.8 ± 2.2	10.7 ± 2.3	10.5 ± 1.9	17.5 ± 3.2	12.4 ± 4.0	9.6 ± 5.1 *∗*	4.9 ± 4.9*∗*	8.8 ± 2.6	9.5 ± 3.7	3.0 ± 2.7*∗*	12.2 ± 4.5	1.7 ± 5.0	187.3 ± 20.2∗∗
720	3.9 ± 3.5∗∗	17.9 ± 4.5∗∗	12.3 ± 5.9∗∗	16.9 ± 3.1	10.9 ± 3.7∗∗	13.7 ± 3.6	9.4 ± 2.7	10.5 ± 2.1	14.9 ± 4.3∗∗	12.9 ± 4.0	8.7 ± 2.8∗∗	7.9 ± 3.2	6.3 ± 3.0*∗*	8.4 ± 2.7	1.1 ± 2.5∗∗	12.5 ± 3.6	1.3 ± 2.8	169.7 ± 15.0∗∗

Females																		
Control	10.8 ± 2.4	12.1 ± 1.5	8.5 ± 3.2	8.6 ± 3.7	8.9 ± 2.9	6.7 ± 2.0	7.1 ± 3.2	6.3 ± 1.7	12.0 ± 5.1	8.2 ± 3.5	7.6 ± 4.0	3.8 ± 3.1	6.3 ± 4.2	4.8 ± 3.0	0.5 ± 3.0	8.5 ± 3.4	-0.6 ± 3.1	120.2 ± 14.5
100	9.1 ± 2.3	11.2 ± 2.6	7.7 ± 3.1	9.1 ± 3.4	8.4 ± 4.7	7.3 ± 3.9	8.5 ± 2.0	6.6 ± 2.8	10.7 ± 4.1	6.0 ± 2.7	7.8 ± 5.1	3.7 ± 3.3	4.9 ± 3.5	5.7 ± 4.2	-1.5 ± 3.5	10.9 ± 5.4	0.3 ± 4.7	116.4 ± 16.7
360	5.3 ± 3.4∗∗	10.8 ± 2.6	7.7 ± 2.3	9.9 ± 1.0	9.3 ± 2.8	7.5 ± 2.8	7.4 ± 3.4	5.7 ± 2.7	13.8 ± 4.0	6.6 ± 2.5	4.7 ± 2.3*∗*	4.8 ± 2.6	4.9 ± 3.2	3.1 ± 3.0	0.7 ± 2.8	6.7 ± 2.6	1.3 ± 3.1	110.2 ± 12.0
720	0.8 ± 3.3∗∗	8.4 ± 3.6∗∗	8.1 ± 2.1	10.3 ± 2.9	8.7 ± 2.5	9.0 ± 1.6∗∗	6.4 ± 1.7	7.0 ± 2.4	8.5 ± 2.5*∗*	7.4 ± 2.0	4.9 ± 2.4*∗*	3.1 ± 2.0	5.1 ± 2.1	2.7 ± 1.8	-1.6 ± 2.7	6.5 ± 2.6	1.8 ± 2.9	97.2 ± 9.0∗∗

*n* = 15 in control and 720 mg/kg bw/day groups; in all other groups *n* = 10.

*∗*p < 0.05, ∗∗p < 0.01, statistical significances were determined with Duncan's multiple range test or with Mann–Whitney *U* test versus control.

**Table:** (b) Summary of mean body weight gain in the recovery period

Group (mg/kg bw/day)	Body weight gain (g) between days				
	89–96	96–103	103–110	110–117	89–117
Males					
Control	12.0 ± 2.7	9.4 ± 3.6	9.2 ± 3.5	5.0 ± 3.1	35.6 ± 3.0
720	8.0 ± 4.1	15.8 ± 4.1*∗*	12.8 ± 5.9	11.4 ± 5.1*∗*	48.0 ± 12.6

Females					
Control	0.6 ± 2.2	3.0 ± 3.5	5.0 ± 1.6	2.2 ± 4.3	10.8 ± 4.4
720	0.2 ± 4.5	2.4 ± 2.3	2.4 ± 7.1	6.4 ± 5.9	11.4 ± 3.8

*n* = 5 for all groups.

*∗*p < 0.05, ∗∗p < 0.01, statistical significances were determined with Duncan's multiple range test or with Mann–Whitney *U* test versus control.

**Table:** (a) Summary of food consumption in the 90-day repeated oral toxicity study

Group (mg/kg bw/day)	Daily mean food consumption (g/animal/d) on weeks												
	1	2	3	4	5	6	7	8	9	10	11	12	13
Males													
Control	20.2 ± 1.60	21.1 ± 1.68	23.1 ± 1.81	22.0 ± 1.50	20.3 ± 1.51	20.4 ± 2.04	18.8 ± 1.62	19.6 ± 1.55	19.4 ± 1.28	17.8 ± 1.60	19.8 ± 1.72	18.0 ± 1.83	16.1 ± 1.52
100	20.5 ± 1.68	20.3 ± 2.98	22.8 ± 3.34	22.1 ± 3.37	20.6 ± 2.74	19.8 ± 2.49	18.7 ± 2.59	17.4 ± 2.09∗∗	18.1 ± 1.96*∗*	16.8 ± 2.02	18.0 ± 1.94*∗*	16.9 ± 1.94	15.3 ± 1.63
360	16.7 ± 1.64∗∗	17.9 ± 1.18∗∗	20.1 ± 1.03∗∗	19.7 ± 1.35∗∗	18.4 ± 1.61*∗*	17.9 ± 2.23*∗*	16.9 ± 2.23*∗*	15.5 ± 1.78∗∗	16.9 ± 1.66∗∗	16.4 ± 1.68	16.7 ± 1.87∗∗	16.2 ± 2.32*∗*	14.0 ± 1.45∗∗
720	14.6 ± 1.17∗∗	16.9 ± 1.19∗∗	18.9 ± 1.21∗∗	18.6 ± 0.81∗∗	17.8 ± 0.93∗∗	17.5 ± 0.82∗∗	16.3 ± 1.29∗∗	15.8 ± 1.09∗∗	16.6 ± 0.91∗∗	16.3 ± 1.16*∗*	16.6 ± 1.14∗∗	15.9 ± 1.39∗∗	13.8 ± 1.36∗∗

Females													
Control	14.3 ± 1.00	13.5 ± 1.07	16.2 ± 1.39	15.8 ± 1.93	15.0 ± 2.64	15.7 ± 2.11	15.0 ± 2.04	15.6 ± 2.17	16.7 ± 2.22	14.6 ± 1.82	16.0 ± 1.95	15.0 ± 1.98	13.6 ± 1.65
100	14.2 ± 1.58	13.3 ± 1.33	14.9 ± 1.36*∗*	15.1 ± 1.38	14.3 ± 1.66	14.0 ± 1.24∗∗	13.5 ± 1.77*∗*	12.9 ± 1.32∗∗	14.2 ± 1.43∗∗	13.5 ± 1.81	14.3 ± 1.86*∗*	13.6 ± 2.07	12.3 ± 2.07
360	11.7 ± 0.81∗∗	11.8 ± 0.92∗∗	14.1 ± 0.88∗∗	14.2 ± 0.83*∗*	13.6 ± 0.97	13.1 ± 1.27∗∗	12.3 ± 1.23∗∗	11.9 ± 1.37∗∗	13.5 ± 1.03∗∗	11.7 ± 1.56∗∗	12.6 ± 1.63∗∗	11.9 ± 1.83∗∗	10.5 ± 1.84∗∗
720	10.4 ± 1.28∗∗	11.5 ± 1.23∗∗	14.0 ± 0.95∗∗	14.1 ± 1.15∗∗	13.1 ± 1.24*∗*	12.7 ± 1.11∗∗	12.0 ± 1.27∗∗	11.3 ± 1.22∗∗	12.5 ± 0.92∗∗	11.3 ± 1.16∗∗	12.0 ± 1.22∗∗	11.7 ± 1.26∗∗	10.1 ± 1.31∗∗

*n* = 15 for control and 720 mg/kg bw/day groups and *n* = 10 for all other groups.

*∗*p < 0.05, ∗∗p < 0.01, statistical significances were determined with Duncan's multiple range test or with Mann–Whitney *U* test versus control.

**Table:** (b) Summary of food consumption in the recovery period

Group (mg/kg bw/day)	Mean food consumption (g/animal/d) on weeks			
	1	2	3	4
Males				
Control	23.9 ± 1.2	25.5 ± 1.2	24.5 ± 1.1	23.9 ± 1.0
720	19.7 ± 1.7∗∗	24.2 ± 1.4	22.6 ± 1.8	22.7 ± 2.0

Females				
Control	15.9 ± 1.1	17.9 ± 1.7	16.2 ± 1.1	16.5 ± 1.1
720	12.8 ± 1.3∗∗	15.4 ± 1.8	13.5 ± 2.5	14.9 ± 0.9*∗*

*n* = 5 for all groups.

*∗*p < 0.05, ∗∗p < 0.01, statistical significances were determined with Duncan's multiple range test or with Mann–Whitney *U* test versus control.

**Table 10:** Summary of selected† hematological findings in the 90-day repeated oral toxicity study.

Group	NEU	LYM	MONO	EOS	RBC	HGB	HCT	MCV	MCH	MCHC	PLT	RET	APTT
(mg/kg bw/day)	%	%	%	%	×10^12^/L	g/L	L/L	fL	pg	g/L	×10^9^/L	%	sec
Male (main groups, *n* = 10 each)													
Control	14.21 ± 2.44	82.56 ± 2.64	2.20 ± 0.40	0.99 ± 0.31	9.46 ± 0.28	167.60 ± 4.99	0.46 ± 0.01	49.06 ± 1.52	17.71 ± 0.43	361.00 ± 4.24	787.30 ± 90.20	3.01 ± 0.44	21.73 ± 3.64
100	19.25 ± 3.80∗∗	77.81 ± 4.17*∗*	2.13 ± 0.38	0.77 ± 0.29	9.49 ± 0.54	166.70 ± 6.73	0.46 ± 0.02	48.97 ± 1.92	17.59 ± 0.59	359.50 ± 5.15	804.00 ± 93.50	3.26 ± 0.33	22.67 ± 1.59
360	16.40 ± 4.50	81.30 ± 4.88	1.85 ± 0.46	0.44 ± 0.20∗∗	9.72 ± 0.47	168.80 ± 6.99	0.47 ± 0.02	47.96 ± 1.47	17.39 ± 0.45	362.30 ± 5.19	848.80 ± 79.82	3.03 ± 0.27	25.48 ± 4.32*∗*
720	18.03 ± 3.98*∗*	79.70 ± 4.19	1.84 ± 0.57	0.42 ± 0.25∗∗	9.41 ± 0.55	158.30 ± 6.85∗∗	0.44 ± 0.02∗∗	46.75 ± 2.25*∗*	16.86 ± 0.59∗∗	360.60 ± 6.17	845.10 ± 58.72	3.17 ± 0.35	22.45 ± 2.56
Historical range*♮*	9.7–38.7	56.5–87.8	1.8–5.1	0.3–9.0	7.52–10.21	141–180	0.404–0.489	45.4–53.7	16.6–18.8	349–379	595–957	2.05–4.65	15.2–31.8

Male (recovery groups, *n* = 5 each)													
Control	15.42 ± 4.77	80.12 ± 5.80	3.06 ± 0.82	1.34 ± 0.49	9.90 ± 0.56	171.60 ± 5.86	0.47 ± 0.02	47.34 ± 2.40	17.34 ± 0.77	367.00 ± 4.64	831.00 ± 91.48	3.39 ± 0.20	19.80 ± 3.67
720	20.94 ± 6.68	75.74 ± 6.45	2.20 ± 0.66	1.08 ± 0.33	9.66 ± 0.44	167.00 ± 7.91	0.46 ± 0.03	47.56 ± 2.04	17.28 ± 0.38	363.80 ± 8.47	758.00 ± 125.59	3.62 ± 0.28	21.02 ± 1.82

Female (main groups, *n* = 10 each)													
Control	11.49 ± 4.30	85.13 ± 4.83	2.06 ± 0.59	1.30 ± 0.54	8.49 ± 0.39	159.80 ± 2.86	0.45 ± 0.01	52.88 ± 2.90	18.87 ± 0.83	356.90 ± 5.47	753.80 ± 94.29	3.95 ± 0.68	21.01 ± 2.26
100	18.80 ± 5.14∗∗	78.45 ± 5.32∗∗	1.93 ± 0.50	0.82 ± 0.53*∗*	8.77 ± 0.25	159.70 ± 5.01	0.45 ± 0.02	50.84 ± 1.75*∗*	18.20 ± 0.46*∗*	358.30 ± 5.52	788.60 ± 63.52	3.70 ± 0.69	23.73 ± 4.91
360	13.99 ± 2.85	83.81 ± 3.08	1.60 ± 0.44*∗*	0.60 ± 0.22∗∗	8.89 ± 0.37*∗*	156.90 ± 5.32	0.44 ± 0.01	49.54 ± 2.02∗∗	17.66 ± 0.67∗∗	356.50 ± 3.31	835.10 ± 73.32*∗*	3.35 ± 0.45*∗*	21.49 ± 2.74
720	14.06 ± 3.28	83.65 ± 3.53	1.47 ± 0.37*∗*	0.80 ± 0.40*∗*	8.99 ± 0.25∗∗	155.30 ± 4.72	0.43 ± 0.01	48.26 ± 0.75∗∗	17.28 ± 0.29∗∗	358.00 ± 5.03	757.50 ± 63.89	3.06 ± 0.47∗∗	20.87 ± 3.40
Historical range*♮*	5.8–33.3	63.4–91.1	1.1–3.9	0.4–2.1	7.71–9.17	148–168	0.415–0.467	47.0–60.1	17.2–20.8	346–372	549–1103	2.77–5.63	17.4–27.9

Female (recovery groups, *n* = 5 each)													
Control	19.62 ± 6.96	77.26 ± 6.93	1.84 ± 0.38	1.22 ± 0.35	8.88 ± 0.32	161.80 ± 5.36	0.45 ± 0.01	50.74 ± 0.94	18.22 ± 0.33	359.60 ± 3.51	817.20 ± 97.76	3.79 ± 0.60	23.94 ± 4.60
720	21.38 ± 5.64	75.60 ± 5.43	1.60 ± 0.74	1.42 ± 0.48	8.99 ± 0.30	160.60 ± 7.23	0.44 ± 0.02	49.30 ± 2.49	17.88 ± 0.81	362.80 ± 2.39	900.00 ± 129.28	3.73 ± 0.62	21.50 ± 5.65

Data represent the mean values and the standard deviation.

†: only parameters with statistically significant findings are shown in table.

*∗P* < 0.05 and ∗∗P < 0.01, statistical significances were determined with Duncan's multiple range test or with Mann–Whitney *U* test versus control.

*♮*: minimum and maximum levels reported as the range of historical control values.

**Table 11:** Summary of selected† clinical chemistry findings in the 90-day repeated oral toxicity study.

Group	ALT	AST	GGT	ALP	TBIL	CREA	CHOL	Pi	ALB	TPROT
(mg/kg bw/day)	U/L	U/L	U/L	U/L	*μ*mol/L	*μ*mol/L	mmol/L	mmol/L	g/L	g/L
Male (main groups, *n* = 10 each)										
Control	65.11 ± 15.73	98.99 ± 17.04	1.21 ± 0.23	129.10 ± 35.04	1.93 ± 0.43	28.84 ± 2.54	1.90 ± 0.22	1.93 ± 0.24	34.81 ± 0.60	61.99 ± 2.53
100	52.96 ± 9.36	95.25 ± 9.39	1.27 ± 0.36	124.80 ± 40.28	1.52 ± 0.25*∗*	27.83 ± 2.98	1.86 ± 0.38	2.26 ± 0.53	35.38 ± 1.15	64.65 ± 3.49
360	62.35 ± 16.31	83.03 ± 6.33*∗*	2.11 ± 0.67∗∗	129.90 ± 43.44	1.70 ± 0.24	29.01 ± 2.29	1.84 ± 0.26	2.38 ± 0.34*∗*	36.11 ± 1.41*∗*	66.90 ± 5.38*∗*
720	51.50 ± 11.56*∗*	88.31 ± 11.96	3.44 ± 1.06∗∗	161.60 ± 38.08	1.87 ± 0.33	29.27 ± 3.56	1.68 ± 0.42	2.10 ± 0.37	36.47 ± 1.78∗∗	67.29 ± 4.40*∗*
Historical range*♮*	28.0–86.2	67.9–135.7	0.0–2.2	56–184	0.71–2.79	18.5 ± 37.2	1.27–2.44	1.40–2.41	33.0–37.4	56.8–68.0

Male (recovery groups, *n* = 5 each)										
Control	57.56 ± 15.48	89.22 ± 11.97	0.98 ± 0.18	104.60 ± 27.83	1.61 ± 0.30	30.42 ± 2.46	2.22 ± 0.29	1.95 ± 0.37	35.38 ± 0.88	66.48 ± 3.33
720	48.70 ± 17.40	76.78 ± 11.47	1.48 ± 0.16∗∗	89.00 ± 16.00	1.78 ± 0.41	30.56 ± 4.48	1.82 ± 0.42	2.08 ± 0.28	36.00 ± 0.99	65.58 ± 3.27

Female (main groups, *n* = 10 each)										
Control	52.32 ± 7.80	85.88 ± 10.45	1.54 ± 0.29	55.80 ± 16.72	1.78 ± 0.22	31.81 ± 2.69	1.94 ± 0.38	1.59 ± 0.23	35.35 ± 1.23	62.16 ± 2.61
100	51.04 ± 10.64	91.17 ± 22.32	1.35 ± 0.47	64.70 ± 26.19	1.91 ± 0.39	30.36 ± 2.30	1.80 ± 0.27	1.96 ± 0.36	36.18 ± 1.50	64.78 ± 2.63
360	51.40 ± 8.64	84.86 ± 12.67	3.11 ± 0.74∗∗	72.70 ± 22.96	1.60 ± 0.25	30.85 ± 3.33	2.19 ± 0.32	1.99 ± 0.35∗∗	36.33 ± 1.21	64.62 ± 2.83
720	58.11 ± 11.93	85.12 ± 15.40	7.41 ± 1.72∗∗	91.10 ± 23.86∗∗	2.13 ± 0.69	30.37 ± 2.35	2.71 ± 0.41∗∗	1.84 ± 0.15∗∗	35.90 ± 1.84	65.15 ± 4.63
Historical range*♮*	23.4–87.7	71.0–141.8	0.0–2.9	25.0–126	1.23–3.30	25.6–40.8	1.35–3.39	0.93–2.01	33.2–41.0	57.1–74.5

Female (recovery groups, *n* = 5 each)										
Control	48.12 ± 13.64	73.58 ± 11.26	1.06 ± 0.15	53.60 ± 13.94	2.03 ± 0.28	34.44 ± 2.50	2.37 ± 0.50	1.39 ± 0.32	37.28 ± 2.70	68.94 ± 5.95
720	38.40 ± 4.83	70.44 ± 10.04	1.96 ± 0.46∗∗	49.60 ± 10.36	1.78 ± 0.24	30.72 ± 2.12*∗*	2.22 ± 0.31	1.22 ± 0.28	36.76 ± 1.09	66.44 ± 1.28

Data represent the mean values and the standard deviation.

†: only parameters with statistically significant findings are shown in table.

*∗P* < 0.05 and ∗∗*P* < 0.01, statistical significances were determined with Duncan's multiple range test or with Mann–Whitney *U* test versus control.

*♮*: minimum and maximum levels reported as the range of historical control values.

**Table 12:** Summary of organ weights (g) in the 90-day repeated oral toxicity study.

Group (mg/kg bw/day)	Body weight	Brain	Liver	Kidneys	Heart	Thymus	Spleen	Testes/uterus	Epididymides/ovaries	Adrenals	Thyroid
Male (main groups, *n* = 10 each)											
Control	383.3 ± 27.86	2.06 ± 0.14	9.65 ± 0.95	2.12 ± 0.18	1.09 ± 0.09	0.40 ± 0.09	0.64 ± 0.11	3.45 ± 0.20	1.68 ± 0.13	0.068 ± 0.008	0.023 ± 0.006
100	374.1 ± 42.85	2.02 ± 0.09	9.70 ± 1.04	2.13 ± 0.27	1.05 ± 0.14	0.35 ± 0.08	0.59 ± 0.07	3.48 ± 0.29	1.59 ± 0.14	0.072 ± 0.008	0.020 ± 0.006
360	345.7 ± 18.90∗∗	2.01 ± 0.11	11.05 ± 0.79∗∗	2.04 ± 0.16	0.94 ± 0.09∗∗	0.31 ± 0.09*∗*	0.55 ± 0.09	3.24 ± 0.27	1.48 ± 0.16∗∗	0.080 ± 0.012*∗*	0.022 ± 0.004
720	329.4 ± 18.86∗∗	2.02 ± 0.12	13.50 ± 1.37∗∗	2.08 ± 0.15	0.95 ± 0.08∗∗	0.29 ± 0.07∗∗	0.55 ± 0.07	3.29 ± 0.25	1.36 ± 0.11∗∗	0.080 ± 0.012*∗*	0.023 ± 0.005
Historical range*♮*	344–488	1.81–2.39	7.95–14.14	1.79–2.73	0.97–1.50	0.23–0.84	0.26–0.96	2.58–4.20	1.39–1.93	0.047–0.097	0.015–0.038

Male (recovery group, *n* = 5 each)											
Control	434.0 ± 16.08	2.07 ± 0.01	10.65 ± 0.44	2.26 ± 0.08	1.02 ± 0.04	0.38 ± 0.07	0.68 ± 0.08	3.50 ± 0.09	1.81 ± 0.09	0.071 ± 0.007	0.025 ± 0.002
720	373.2 ± 19.25∗∗	2.05 ± 0.06	8.93 ± 0.81∗∗	2.19 ± 0.09	0.99 ± 0.03	0.36 ± 0.07	0.63 ± 0.08	3.51 ± 0.29	1.71 ± 0.08	0.063 ± 0.003*∗*	0.026 ± 0.003

Female (main groups, *n* = 10 each)											
Control	239.0 ± 18.90	1.97 ± 0.12	6.42 ± 0.40	1.49 ± 0.16	0.77 ± 0.07	0.30 ± 0.04	0.55 ± 0.10	0.79 ± 0.23	0.159 ± 0.025	0.083 ± 0.013	0.023 ± 0.005
100	234.6 ± 15.97	1.88 ± 0.15	6.70 ± 0.56	1.43 ± 0.09	0.76 ± 0.05	0.37 ± 0.05∗∗	0.46 ± 0.06∗∗	0.59 ± 0.13*∗*	0.145 ± 0.041	0.084 ± 0.014	0.020 ± 0.005
360	224.3 ± 13.14*∗*	1.91 ± 0.13	7.40 ± 0.60∗∗	1.34 ± 0.13*∗*	0.71 ± 0.08	0.33 ± 0.06	0.43 ± 0.06∗∗	0.50 ± 0.20∗∗	0.141 ± 0.025	0.093 ± 0.013	0.020 ± 0.005
720	213.4 ± 11.75∗∗	1.88 ± 0.07	9.21 ± 0.54∗∗	1.36 ± 0.10*∗*	0.69 ± 0.06*∗*	0.30 ± 0.03	0.36 ± 0.05∗∗	0.46 ± 0.12∗∗	0.140 ± 0.037	0.100 ± 0.012*∗*	0.023 ± 0.009
Historical range*♮*	206–285	1.75–2.18	5.30–7.97	1.23–2.00	0.66–0.96	0.24–0.54	0.33–0.75	0.40–2.07	0.098–0.208	0.063–0.113	0.012–0.029

Female (recovery group, *n* = 5 each)											
Control	248.4 ± 8.73	2.04 ± 0.05	6.63 ± 0.45	1.57 ± 0.11	0.74 ± 0.05	0.38 ± 0.05	0.49 ± 0.04	0.72 ± 0.10	0.139 ± 0.030	0.071 ± 0.009	0.022 ± 0.002
720	217.2 ± 9.58∗∗	1.92 ± 0.03∗∗	5.86 ± 0.21∗∗	1.37 ± 0.07∗∗	0.67 ± 0.04*∗*	0.33 ± 0.03	0.43 ± 0.04*∗*	0.48 ± 0.10∗∗	0.130 ± 0.031	0.075 ± 0.005	0.024 ± 0.002

Data represent the mean values and the standard deviation.

*∗P* < 0.05 and ∗∗ *P* < 0.01, statistical significances were determined with Duncan's multiple range test or with Mann–Whitney *U* test versus control.

*♮*: minimum and maximum levels reported as the range of historical control values.

**Table 13:** Summary of relative organ weights (organ weight relative to body weight) (%) in the 90-day repeated oral toxicity study.

Group (mg/kg bw/day)	Brain	Liver	Kidneys	Heart	Thymus	Spleen	Testes/uterus	Epididymides/ovaries	Adrenals	Thyroid
Male (main groups, *n* = 10 each)										
Control	0.539 ± 0.028	2.515 ± 0.098	0.554 ± 0.036	0.284 ± 0.018	0.103 ± 0.021	0.165 ± 0.026	0.902 ± 0.051	0.441 ± 0.052	0.018 ± 0.002	0.006 ± 0.002
100	0.545 ± 0.054	2.595 ± 0.081	0.570 ± 0.040	0.281 ± 0.034	0.094 ± 0.012	0.157 ± 0.011	0.933 ± 0.054	0.426 ± 0.038	0.019 ± 0.003	0.005 ± 0.002
360	0.581 ± 0.043	3.196 ± 0.147∗∗	0.591 ± 0.044*∗*	0.272 ± 0.019	0.090 ± 0.024	0.158 ± 0.025	0.938 ± 0.086	0.430 ± 0.049	0.023 ± 0.004∗∗	0.006 ± 0.001
720	0.617 ± 0.055∗∗	4.093 ± 0.228∗∗	0.631 ± 0.027∗∗	0.290 ± 0.028	0.088 ± 0.020	0.168 ± 0.021	1.002 ± 0.080∗∗	0.414 ± 0.026	0.024 ± 0.004∗∗	0.007 ± 0.002
Historical range*♮*	0.441–0.599	1.916–3.108	0.466–0.650	0.216–0.311	0.057–0.191	0.058–0.219	0.642–1.011	0.320–0.512	0.012–0.023	0.0037–0.0089

Male (recovery group, *n* = 5 each)										
Control	0.478 ± 0.015	2.454 ± 0.089	0.522 ± 0.019	0.236 ± 0.015	0.087 ± 0.015	0.158 ± 0.019	0.806 ± 0.014	0.418 ± 0.027	0.017 ± 0.002	0.006 ± 0.000
720	0.550 ± 0.032∗∗	2.392 ± 0.158	0.587 ± 0.037∗∗	0.265 ± 0.020*∗*	0.098 ± 0.021	0.168 ± 0.023	0.941 ± 0.082∗∗	0.460 ± 0.032	0.017 ± 0.001	0.007 ± 0.000∗∗

Female (main groups, *n* = 10 each)										
Control	0.825 ± 0.028	2.693 ± 0.174	0.625 ± 0.040	0.322 ± 0.022	0.128 ± 0.018	0.230 ± 0.034	0.327 ± 0.088	0.0669 ± 0.0110	0.0348 ± 0.0043	0.0096 ± 0.0022
100	0.805 ± 0.072	2.854 ± 0.133	0.609 ± 0.024	0.324 ± 0.027	0.159 ± 0.024∗∗	0.196 ± 0.023∗∗	0.251 ± 0.051*∗*	0.0616 ± 0.0162	0.0357 ± 0.0051	0.0083 ± 0.0018
360	0.853 ± 0.094	3.298 ± 0.144∗∗	0.598 ± 0.034	0.317 ± 0.033	0.145 ± 0.026	0.191 ± 0.023∗∗	0.224 ± 0.094∗∗	0.0627 ± 0.0108	0.0413 ± 0.0054*∗*	0.0091 ± 0.0027
720	0.882 ± 0.055*∗*	4.322 ± 0.281∗∗	0.637 ± 0.038	0.323 ± 0.025	0.141 ± 0.017	0.169 ± 0.018∗∗	0.215 ± 0.055∗∗	0.0653 ± 0.0163	0.0469 ± 0.0068∗∗	0.0109 ± 0.0037
Historical range*♮*	0.681–0.943	2.172–3.214	0.530–0.752	0.273–0.396	0.093–0.217	0.140–0.298	0.167–0.852	0.043–0.086	0.026–0.044	0.0052–0.0128

Female (recovery group, *n* = 5 each)										
Control	0.820 ± 0.023	2.671 ± 0.167	0.633 ± 0.032	0.299 ± 0.012	0.151 ± 0.020	0.196 ± 0.020	0.290 ± 0.045	0.0562 ± 0.0137	0.0285 ± 0.0031	0.0090 ± 0.0004
720	0.885 ± 0.043*∗*	2.700 ± 0.064	0.629 ± 0.014	0.309 ± 0.016	0.152 ± 0.008	0.197 ± 0.023	0.221 ± 0.047*∗*	0.0600 ± 0.0136	0.0345 ± 0.0027*∗*	0.0109 ± 0.0010∗∗

Data represent the mean values and the standard deviation.

*∗P* < 0.05 and ∗∗*P* < 0.01, statistical significances were determined with Duncan's multiple range test or with Mann–Whitney *U* test versus control.

*♮*: minimum and maximum levels reported as the range of historical control values.

**Table 14:** Summary of relative organ weights (organ and body weight relative to brain weight) (%) in the 90-day repeated oral toxicity study.

Group (mg/kg bw/day)	Body weight	Liver	Kidneys	Heart	Thymus	Spleen	Testes/uterus	Epididymides/ovaries	Adrenals	Thyroid
Male (main groups, *n* = 10 each)										
Control	18591.6 ± 968.39	468.24 ± 40.18	102.89 ± 6.65	52.69 ± 3.69	19.15 ± 3.63	30.70 ± 4.40	167.49 ± 8.20	81.70 ± 8.27	3.31 ± 0.39	1.09 ± 0.28
100	18490.1 ± 1785.06	479.69 ± 46.21	105.21 ± 11.56	51.68 ± 5.97	17.48 ± 3.71	28.94 ± 3.10	172.03 ± 13.70	78.46 ± 6.78	3.57 ± 0.45	1.01 ± 0.34
360	17293.5 ± 1365.76	552.65 ± 49.26∗∗	102.35 ± 11.99	46.98 ± 5.17*∗*	15.64 ± 4.23	27.28 ± 4.46	161.87 ± 16.34	74.05 ± 7.67*∗*	4.02 ± 0.61∗∗	1.11 ± 0.22
720	16340.7 ± 1594.47∗∗	670.06 ± 90.43∗∗	103.09 ± 10.65	47.16 ± 3.98*∗*	14.29 ± 3.43*∗*	27.46 ± 4.11	163.26 ± 16.05	67.57 ± 6.46∗∗	3.96 ± 0.50∗∗	1.14 ± 0.25
Historical range*♮*	16681–22689	371.50–660.20	91.59–131.25	43.53–65.22	10.45–38.18	11.82–41.71	129.65–186.05	63.76–98.40	2.19–4.32	0.78–1.80

Male (recovery group, *n* = 5 each)										
Control	20923.4 ± 682.77	513.36 ± 19.85	109.06 ± 3.33	49.28 ± 2.09	18.12 ± 3.19	32.97 ± 3.96	168.56 ± 3.71	87.37 ± 4.30	3.44 ± 0.34	1.19 ± 0.07
720	18234.7 ± 1067.39∗∗	435.79 ± 31.87∗∗	106.81 ± 1.71	48.18 ± 2.34	17.79 ± 3.54	30.55 ± 3.78	171.55 ± 16.96	83.77 ± 5.21	3.07 ± 0.23	1.25 ± 0.14

Female (main groups, *n* = 10 each)										
Control	12135.2 ± 419.40	326.63 ± 20.94	75.83 ± 6.20	39.04 ± 3.11	15.47 ± 2.16	27.91 ± 3.98	39.68 ± 10.63	8.11 ± 1.30	4.22 ± 0.51	1.16 ± 0.27
100	12513.6 ± 1162.30	357.88 ± 44.84	76.19 ± 6.93	40.48 ± 4.55	19.89 ± 3.29∗∗	24.50 ± 3.34*∗*	31.50 ± 6.98	7.74 ± 2.23	4.50 ± 0.95	1.04 ± 0.25
360	11848.4 ± 1337.88	391.14 ± 50.90∗∗	70.90 ± 9.89	37.63 ± 6.71	17.29 ± 4.20	22.74 ± 4.47∗∗	26.75 ± 12.08∗∗	7.46 ± 1.70	4.89 ± 0.79	1.06 ± 0.23
720	11377.7 ± 735.76*∗*	490.87 ± 33.83∗∗	72.47 ± 6.17	36.65 ± 3.17	15.97 ± 2.06	19.19 ± 2.33∗∗	24.65 ± 6.98∗∗	7.41 ± 1.83	5.32 ± 0.70∗∗	1.24 ± 0.44
Historical range*♮*	10600.0–14685.7	276.04–412.95	65.08–104.71	33.65–49.74	11.82–27.55	17.84–38.34	18.96–99.52	5.19–10.79	3.12–5.49	0.65–1.53

Female (recovery group, *n* = 5 each)										
Control	12200.5 ± 339.93	325.87 ± 21.65	77.26 ± 4.22	36.44 ± 2.25	18.49 ± 2.75	23.86 ± 1.79	35.34 ± 4.81	6.84 ± 1.58	3.47 ± 0.38	1.10 ± 0.06
720	11316.1 ± 560.61*∗*	305.45 ± 14.34	71.15 ± 3.85*∗*	35.03 ± 2.81	17.19 ± 1.42	22.18 ± 1.86	24.95 ± 5.06*∗*	6.78 ± 1.55	3.90 ± 0.25	1.23 ± 0.11

Data represent the mean values and the standard deviation.

*∗P* < 0.05 and ∗∗*P* < 0.01, statistical significances were determined with Duncan's multiple range test or with Mann–Whitney *U* test versus control.

*♮*: minimum and maximum levels reported as the range of historical control values.

**Table 15:** Summary of notable histopathology findings in the 90-day repeated oral toxicity study.

Organs	Observations	Dose groups (mg/kg bw/day)					
		Control		100	360	720	
		Main	Recovery			Main	Recovery
Male							
Adrenal gland	Increased cytoplasmic vacuolation	0/10	0/5	0/10	0/10	6/10	0/5
Kidney	Cyst	1/10	0/5	0/1	/	0/10	0/5
	Mineral deposits	0/10	0/5	1/1	/	0/10	0/5
Lungs	Alveolar emphysema	0/10	0/5	/	/	1/10	1/5
	Alveolar histiocytosis	2/10	0/5	/	/	1/10	0/5

Female							
Adrenal gland	Increased cytoplasmic vacuolation	0/10	0/5	0/10	0/10	8/10	0/5
Kidneys	Focal fibrosis	0/10	0/5	1/1	0/1	0/10	0/5
Lungs	Alveolar emphysema	1/10	0/5	/	/	0/10	1/5
	Acute hemorrhage	0/10	1/5	/	/	0/10	0/5
	Alveolar histiocytosis	1/10	0/5	/	/	3/10	0/5
Uterus	Dilatation	4/10	3/5	/	/	2/10	1/5

/, not examined.

Data represent the number of animals with observation per number of animals observed.

## Abbreviations

2-AA: 2-Aminoanthracene
9-AA: 9-Aminoacridine
ANOVA: Analysis of Variance
bw: Body weight
CBD: Cannabidiol
CREA: Creatinine
DMSO: Dimethyl sulfoxide
EC: European Commission
EPA: Environmental Protection Agency
OPPTS: Office of Prevention, Pesticides and Toxic Substances
FOB: Functional observation battery
GGT: Gamma-glutamyl transferase
ICH: International Conference on Harmonisation
MMS: Methyl methanesulfonate
MPCE: Micronucleated polychromatic erythrocytes
NOAEL: No observed adverse effect level
NPD: 4-Nitro-1,2-phenylenediamine
OECD: Organisation for Economic Cooperation and Development
PCE: Polychromatic erythrocytes
SAZ: Sodium azide
THC: Delta-9-tetrahydrocannabinol.
